# Impact of Ketogenic and Mediterranean Diets on Gut Microbiota Profile and Clinical Outcomes in Drug-Naïve Patients with Diabesity: A 12-Month Pilot Study

**DOI:** 10.3390/metabo15010022

**Published:** 2025-01-06

**Authors:** Vanessa Palmas, Andrea Deledda, Vitor Heidrich, Giuseppina Sanna, Giulia Cambarau, Michele Fosci, Lorenzo Puglia, Enrico Antonio Cappai, Alessio Lai, Andrea Loviselli, Aldo Manzin, Fernanda Velluzzi

**Affiliations:** 1 Department of Biomedical Sciences, University of Cagliari, 09042 Monserrato, Italy; vanessa.palmas@unica.it (V.P.); g.sanna@unica.it (G.S.); 2 Obesity Unit, Department of Medical Sciences and Public Health, University of Cagliari, 09124 Cagliari, Italy; andredele@tiscali.it (A.D.); giuliacambarau@tiscali.it (G.C.); cappai42@gmail.com (E.A.C.); fernanda.velluzzi@unica.it (F.V.); 3 Departamento de Bioquímica, Instituto de Química, Universidade de São Paulo, São Paulo 05508-900, Brazil; vheidrich@mochsl.org.br; 4 Centro de Oncologia Molecular, Hospital Sírio-Libanês, São Paulo 01308-050, Brazil; 5 Endocrinology Unit, Department of Medical Sciences and Public Health, University of Cagliari, 09042 Monserrato, Italy; michele.fosci@aslsulcis.it (M.F.); lorenzo.puglia02@icatt.it (L.P.); alovise2@gmail.com (A.L.); 6 Diabetologia, P.O. Binaghi, ASSL Cagliari, 09126 Cagliari, Italy; alexlai1@tiscali.it

**Keywords:** very-low-calorie ketogenic diet, Mediterranean diet, type 2 diabetes mellitus, obesity, diabesity, glucometabolic status, patient-centered approach, lifestyle, gut microbiota, 16S rRNA

## Abstract

**Background/Objectives**: Managing type 2 diabetes mellitus (T2DM) and obesity requires a multidimensional, patient-centered approach including nutritional interventions (NIs) and physical activity. Changes in the gut microbiota (GM) have been linked to obesity and the metabolic alterations typical of T2DM and obesity, and they are strongly influenced by diet. However, few studies have evaluated the effects on the GM of a very-low-calorie ketogenic diet (VLCKD) in patients with T2DM, especially in the mid-term and long-term. This longitudinal study is aimed at evaluating the mid-term and long-term impact of the VLCKD and Mediterranean diet (MD) on the GM and on the anthropometric, metabolic, and lifestyle parameters of 11 patients with T2DM and obesity (diabesity). This study extends previously published results evaluating the short-term (three months) impact of these NIs on the same patients. **Methods**: At baseline, patients were randomly assigned to either a VLCKD (KETO group) or a Mediterranean diet (MEDI group). After two months, the KETO group gradually shifted to a Mediterranean diet (VLCKD-MD), according to current VLCKD guidelines. From the fourth month until the end of the study both groups followed a similar MD. Previous published results showed that VLCKD had a more beneficial impact than MD on several variables for 3 months of NI. In this study, the analyses were extended until six (T6) and twelve months (T12) of NI by comparing data prospectively and against baseline (T0). The GM analysis was performed through next-generation sequencing. **Results**: Improvements in anthropometric and metabolic parameters were more pronounced in the KETO group at T6, particularly for body mass index (−5.8 vs. −1.7 kg/m^2^; *p* = 0.006) and waist circumference (−15.9 vs. −5.2 cm; *p* = 0.011). At T6, a significant improvement in HbA1c (6.7% vs. 5.5% *p* = 0.02) and triglyceride (158 vs. 95 mg/dL *p* = 0.04) values compared to T0 was observed only in the KETO group, which maintained the results achieved at T3. The VLCKD-MD had a more beneficial impact than the MD on the GM phenotype. A substantial positive modulatory effect was observed especially up to the sixth month of the NI in KETO due to the progressive increase in bacterial markers of human health. After the sixth month, most markers of human health decreased, though they were still increased compared with baseline. Among them, the Verrucomicrobiota phylum was identified as the main biomarker in the KETO group, together with its members Verrucomicrobiae, Akkermansiaceae, Verrucomicrobiales, and *Akkermansia* at T6 compared with baseline. **Conclusions**: Both dietary approaches ameliorated health status, but VLCKD, in support of the MD, has shown greater improvements on anthropometric and metabolic parameters, as well as on GM profile, especially up to T6 of NI.

## 1. Introduction

Type 2 diabetes mellitus (T2DM) is a chronic and progressive disease with a growing prevalence, usually associated with overweight outcomes and obesity, particularly with visceral obesity [1,2].

T2DM is characterized by a reduced biological response to the hypoglycemic effects of insulin and an inadequate insulin secretion, which leads to a hyperglycemic condition, the main feature of this disease [3], and a low-grade inflammation [4]. T2DM increases the morbidity and mortality of cardiovascular, renal, infectious, and oncologic diseases [5]. Moreover, a higher prevalence of psychopathological disorders, especially anxiety and depression, has been reported among patients with T2DM and metabolic syndrome [6]. Indeed, T2DM is a stressful factor for the body, and it can lead to a reduced life expectancy if left untreated for many years.

It is estimated that every decade, T2DM corresponds to a reduction in life expectancy by about 3–4 years [7].

T2DM is linked to both modifiable and unmodifiable risk factors, such as genetic predisposition [8], and is closely connected to lifestyle [9]. It is well established that poor dietary habits, particularly the consumption of added sugars and low-quality fats, along with physical inactivity and sedentary behaviors, are major contributors to T2DM and visceral obesity [10,11,12,13]. In particular, a chronic positive energy balance drives weight gain and excess visceral adipose tissue, promoting metabolic alterations that impair insulin secretion and its effectiveness [14]. Beyond insulin, the action of other hormones (glucagon, cortisol, and growth hormone), known for their counter-regulatory action, is also altered [15]. Other emerging causes of T2DM linked to nutrition include low economic status, low quality diet, and food insecurity, which reduce the ability to buy healthy foods like vegetables and fruits [16], which are known for their positive effects on health and anti-inflammatory properties [17,18]. Providing healthy meals can help improve metabolic status [19].

In addition to lifestyle, other factors can contribute to T2DM pathogenesis, such as altered circadian rhythms [20] and sleep [21], stress [22], smoking [23], endocrine disruptors [24], some drugs [25], and COVID-19 infection [26].

Furthermore, increasing evidence shows that the microbiota, particularly the gut microbiota (GM), represented by the microbial communities residing in the gut, can be a contributing factor to obesity and T2DM [27].

The GM has a symbiotic relationship with its host, influencing immunity, inflammation, the aging process, drug response, energy metabolism, and the synthesis of biomolecules derived from food [28]. Current research shows that the GM may influence biological processes through several mechanisms, including the production of bioactive metabolites such as short-chain fatty acids, the modulation of bile acid metabolism, and cross-talk with the immune system. These mechanisms may negatively affect insulin sensitivity and secretion and have been linked to the development of T2DM [29]. The modulation of calorie extraction from diet by the GM is another factor potentially implicated in the pathogenesis of obesity and T2DM. On the other hand, diet is one of the main factors modulating the GM, influencing its diversity and composition [29]. An altered GM profile appears to be an essential link between overweight and metabolic abnormalities [30]. However, defining both the normal GM pattern and the status of dysbiosis, an alteration in the GM composition that may favor various diseases, such as cardiovascular, respiratory, inflammatory, metabolic, and neoplastic diseases, is not simple [29,31].

### Nutritional Treatment of T2DM

The nutritional management of T2DM is a highly debated topic. Modern guidelines recommend a holistic, patient-centered approach, tailored to the individual needs of each patient [32]. This approach involves personalizing both nutrition and physical activity, prescribing medications when necessary and, in case of management difficulties, considering bariatric surgery [33,34]. In terms of the nutritional approach, scientific societies recommend that practitioners prescribe a Mediterranean diet (MD), which includes a wide variety of foods without strict restrictions. Said diet reduces the frequency of consumption of refined grains, packaged foods, sweets, and some animal-based foods like red and processed meat. It allows a moderate intake of white meat, dairy, and seafood and encourages the consumption of plant-based foods, mainly in their unprocessed form, such as whole grains, legumes, nuts, fruits, and vegetables [35,36]. From a clinical perspective, the MD represents the most beneficial eating pattern for preventing and managing T2DM. Moreover, robust evidence links this dietary model to a reduced cardiovascular risk and lower mortality rates even in people with T2DM [36]. However, the outcomes associated with good adherence to the MD also depend on cultural, socioeconomic, or genetic factors [10].

Nevertheless, in recent years, some Italian and European scientific societies have considered the ketogenic diet, in its very-low-calorie form (VLCKD), as a therapeutic option for the treatment of obesity and T2DM (accessed 15 October 2023) [37,38,39,40,41].

The use of VLCKD is suggested only in selected cases and with specific recommendations, such as monitoring the patient after the ketogenic period according to the American Diabetes Association (ADA) guidelines [42], while the European Association for the Study of Diabetes (EASD) recommends against this type of diet [43]. However, such recommendation is based on potential safety concerns arising from inadequate patient selection [44,45].

Diet, including macronutrient composition, fiber intake, prebiotics, probiotics, or additives in foods, is the most important factor in GM modulation. [46]. Particularly, dietary patterns may reciprocally influence the relationship between the GM and health outcomes, including the development and progression of T2DM [46].

The MD and KD, like any other dietary approach, can have specific influences on GM [47,48]. However, there are still few studies that have evaluated the effects of VLCKD for the treatment of obesity and T2DM on the GM, particularly in the long-term [49,50]. Due to the limited number of studies on this topic, we performed a 12-month intervention study comparing the effects of a VLCKD protocol and a moderately hypocaloric MD protocol on the GM of a small sample of patients with recently diagnosed T2DM and obesity. We also evaluated the effects on body composition, metabolic status, lifestyle, and quality of life. The short-term results showed a significant increase in some beneficial microbial taxa, such as the Verrucomicrobiota phylum and its members, particularly *Akkermansia*, after three months on the VLCKD, but not after the MD. Additionally, there was a reduction in microbial taxa associated with obesity and metabolic diseases following the VLCKD. Moreover, the VLCKD had a more significant effect on anthropometric indices and quality of life than the MD. Specifically at T3, the VLCKD resulted in significantly greater weight loss and fat mass reduction compared to the MD, but it also led to a significant reduction in fat-free mass.

After the first two months, patients on the VLCKD gradually shifted to a MD, following a similar protocol as the other group (VLCKD-MD). In the present study, we extended the evaluation of the effects of these nutritional approaches on the same parameters previously assessed for up to 12 months, when all patients were following the MD [51].

## 2. Materials and Methods

### 2.1. Study Design and Characteristics of the Sample

We extended the analysis of our previously published prospective study, which evaluated the short-term (three month) impact of ketogenic (KETO group) and Mediterranean (MEDI group) dietary models on GM modulation, as well as on anthropometric, clinical, and metabolic parameters, in a cohort of patients with obesity or overweight who were recently diagnosed with T2DM. In this follow-up study, we analyzed the same parameters over a 12-month period in the same patients. The study included 11 patients at baseline aged between 45 and 65 years, recruited from the Obesity Unit of the AOU of Cagliari (Italy) and the Diabetology Unit of the PO Binaghi (ASSL of Cagliari, Italy) after a new diagnosis of T2DM. However, due to the COVID-19 infection, some patients were excluded from the follow-up evaluations. Specifically, of the 11 patients enrolled at baseline who followed the treatment until T3 (6 KETO patients and 5 MEDI patients), 1 patient was excluded between T3 and T6, and another 2 patients were excluded between T6 and T12. Therefore, 10 patients completed the nutritional treatment until the end of T6 (5 KETO patients and 5 MEDI patients), and 8 patients adhered to the protocol until the end of T12 (4 KETO patients and 4 MEDI patients). For the mid-term (T6) and long-term (T12) follow-ups, the samples were collected and analyzed after six months (T6) and after twelve months (T12) of each nutritional intervention, and the statistical comparisons of the data were carried out for each time point prospectively and with baseline (T0). The inclusion criteria were newly diagnosed, uncomplicated T2DM (according to IDF/ADA criteria) [2,52], a glycosylated hemoglobin (HbA1c) value of 6.5–8.9%, a body mass index (BMI) ≥ 28 kg/m^2^, and being drug-naïve for T2DM.

The exclusion criteria were the presence of T1DM, serious heart diseases, severe and/or uncontrolled hypertension, severe or uncompensated kidney, liver or thyroid diseases, painful pathologies with severe functional limitations, tumors in chemo/radiotherapy, severe psychopathology, gastrointestinal diseases, therapy with corticosteroids, proton pump inhibitors, antimicrobials, prebiotic or probiotic intake, and any dietary supplements or participation in other dietary regimes in the three months preceding the sample collection [37,38].

After the enrollment and for each time point, the patients underwent a multidimensional evaluation including clinical, metabolic, anthropometric, lifestyle, and a quality of life assessment. At the same time, the analysis of GM, and of its functional activity, was performed at each time point (T6 and T12) by comparing data prospectively and with baseline (T0).

Patients were then randomly assigned to a specific nutritional intervention (NI), with 5 patients (3M, 2F) being assigned to a low-calorie MD according to ADA guidelines (MEDI group) [36] and 6 patients (3M, 3F) to a VLCKD (KETO group) [39].

### 2.2. Dietary Protocols

The two different nutritional protocols, previously described, were followed by the two groups (MEDI and KETO) from baseline until the end of the third month [51]. The VLCKD protocol was structured as a multiphasic protocol, starting with an active phase lasting the first two months and characterized by a state of ketosis which consisted of the exclusive use of protein meal replacement products (Therascience, Monaco, Principality of Monaco) during the first month and natural protein foods during the second month. The total daily energy intake was approximately 800 kcal, with a daily carbohydrate intake of less than 30 g and a daily protein intake set at 1.2–1.5 g/kg of the ideal body weight. Additionally, the ketosis phase included at least two daily servings of non-starchy vegetables and the prescription of omega-3, multivitamins, and multimineral supplements. The third month was characterized by a transition phase in which various carbohydrate food groups were gradually reintroduced. The ketosis status was monitored by measuring blood levels of β-hydroxybutyrate (BOHB) through a finger-prick test and a handheld β-ketone analyzer (Menarini Areo, Florence, Italy) until the third month.

The MD protocol was characterized by a moderate daily energy deficit of 300–500 kcal, not lower than the resting metabolic rate (RMR), with a balanced macronutrient intake that included various isocaloric choices across different food groups (45–50% carbohydrates, 20% proteins, and 30–35% fats). The protocol also recommended at least five daily servings of fruits and vegetables, with an emphasis on varying food choices and favoring those typical of the Mediterranean pattern. At the fourth month, the MEDI group continued the same moderately hypocaloric and balanced MD assigned at the beginning of the study. Meanwhile, the KETO group, after gradually and progressively reintroducing carbohydrates during the third month, increased their carbohydrate intake to match that of the MEDI group (VLCKD-MD). Specifically, starchy foods (2 portions/day) and fruits (2 portions/day) were increased until the participants reached a moderately low-carb and low-calorie diet to allow for a maintenance hypocaloric regimen. Carbohydrate intake ranged from 120 to 180 g per day, and calorie intake ranged from 1400 to 1800 calories per day, adjusted according to patients’ anthropometric characteristics and EASO guidelines. Supplements were progressively discontinued [41].

Therefore, from the fourth to the twelfth month, both groups followed a similar moderately hypocaloric MD. Compliance with the diets was monitored monthly through brief interviews with the nutritionist who also conducted anthropometric measurements. A complete nutritional evaluation, including a food diary and a questionnaire assessing adherence to the MD, was performed at six and twelve months.

### 2.3. Clinical, Metabolic, Anthropometric, Lifestyle, and Health Status Evaluation

At T6 and T12, all patients underwent a clinical examination by the same endocrinologist, which included standardized measurements of blood pressure (BP) and heart rate (HR), an assessment of metabolic status, particularly glucometabolic status, and an evaluation of quality of life.

BP was measured three times after 10 min of sitting at rest using an automatic sphygmomanometer (Omron IntelliSense OMRON HEALTHCARE Co., Ltd., Kyoto, Japan). The reported values represent the average of the last two values.

The metabolic assessment consisted of a 12 h fasting blood sample for determination with standard methods of evaluating fasting plasma glycemia (FPG), glycosylated hemoglobin (HbA1c), C peptide, total cholesterol, HDL (LDL calculated by Friedewald’s formula), total triglycerides, creatininemia, aspartate aminotransferase (AST), alanine aminotransferase (ALT), uricemia, blood count, protein electrophoresis, sodium (Na+), potassium (K+), calcium (Ca2), and a urine test.

The anthropometric evaluation, including measurements of weight, height, body mass index (BMI), and waist circumference (WC), was performed monthly by the same expert nutritionist following current standards [53], as previously described [51].

The analysis of body composition (BIA) at T6 and T12 was carried out using the bioelectrical impedance analyzer BIA-Dex^®^ (Mascaretti, Ancona, Italy). The exam was performed as suggested by the European Society of Parenteral and Enteral Nutrition (ESPEN) [54]. The data obtained corresponded with the resistance and reactance, which, when inserted in the software, indicated fat mass (FM%), fat-free mass (FFM kg), and phase angle (phA °).

The lifestyle evaluation, conducted by the same expert nutritionist during an individual interview, included assessments of nutrition and physical activity levels at both T6 and T12. Briefly, the nutritional assessment consisted of administering the standardized and validated Mediterranean diet score, (MDS, range 0–55) questionnaire [47] previously described [55] and analyzing the food diary for three days of the weekend preceding the visit using the software Winfood^®^ (Medimatica, Colonnella, Italy). The measurement of blood ketone levels, through a finger-prick test, was discontinued at the end of the ketogenic phase (T3).

The physical activity level (PAL) was assessed using the short version of the international physical activity questionnaire (IPAQ), which also evaluates the degree of sedentariness, measured as sitting time (h/day) [56].

The assessment of quality of life and perceived health status was performed using the standardized SF-36 questionnaire, which evaluates eight aspects of physical and mental health. The results (scores ranging from 0 to 100) are reported as two summary scores: the physical component summary (PCS) score and the mental component summary (MCS) score [57].

### 2.4. Gut Microbiota Analysis

#### 2.4.1. Sample Collection

A stool sample was collected from each patient at each time point (at the sixth and twelfth months of nutritional intervention). The stool samples from each patient were self-provided and brought to the laboratory within 3 h. Fresh samples were aliquoted and stored at −80 °C until further processing.

#### 2.4.2. Genomic DNA Extraction, Bacterial DNA Quantification, and 16S Libraries Preparation and Sequencing

Genomic DNA extraction from stool samples and the quantification of bacterial DNA were performed according to the protocols described in the “DNA extraction and quantification” and “Real-time quantitative PCR” sections of the Supplementary 1 document of a previous study, respectively [58]. The protocol of library preparation has been described in detail elsewhere [27]. Libraries of 16S barcoded amplicon were generated using primers targeting the V3–V4 hypervariable region of the bacterial 16S rRNA gene with the Nextera XT index kit (Illumina, Inc., San Diego, CA, USA), and their size and quality were verified using a D1000 reagents kit (Agilent Technologies, Santa Clara, CA, USA) on the Tapestation 4200 system (Agilent Technologies, Santa Clara, CA, USA). The quantification, normalization, pooling of 16S libraries, and the concentration of the PhiX control have been described elsewhere [51]. Combined 16S library and PhiX control were further denatured and sequenced on the MiSeq platform (Illumina) on a v3 paired-end run (300 × 2 cycles) using a MiSeq v3 reagent kit (Illumina).

### 2.5. Bioinformatic and Statistical Analysis

The comparison of anthropometric, metabolic, lifestyle, and health status data before and after NI in each group was performed using a *t*-test for paired data, while a *t*-test for independent samples was performed to compare the mean difference in the same variables between the two groups; a *p*-value (*p*) < 0.05 was considered statistically significant. Regarding GM data and reads processing, the taxonomic assignment and the functional prediction profiles from taxonomic data were performed as previously described [51]. All microbiome analyses (plotting and statistics) were performed in R, and all plots were built with the R package ggplot2, version 3.5.1 [59]. To account for different sequencing depths, samples were normalized to 22,173 reads (the lowest number of reads per sample) via scaling with ranked subsampling (SRS) [60] using the SRS R package, version 0.2.3 [61]. Alpha diversity was assessed considering the ASV richness (observed ASVs), the Shannon index, and the Pielou’s J index, as previously described [51]. Alpha diversity differences between the two nutritional groups at each time point were assessed using the Mann–Whitney U test; alpha diversity differences across time points within each nutritional group were assessed using the Wilcoxon paired test. Beta diversity based on the Bray–Curtis distance matrix and the unweighted and weighted UniFrac metrics was assessed as previously described [51]. It was compared between the two different nutritional groups at each time point and between each time point within the same nutritional intervention group using PCoAs and the PERMANOVA test. The Generalized Linear Mixed-effects Model implemented in MaAsLin2 [62] was used to find significant longitudinal changes in bacterial taxa abundances (from phylum down to a species level) during each diet. The patient was considered a random effect. The statistical significance was tested considering *p* ≤ 0.05, with a Benjamini–Hochberg (BH) correction cut-off at *q* ≤ 0.25. The association between the relative abundance of significant taxonomic levels and clinical and nutritional parameters was evaluated by calculating the Spearman’s correlation in GraphPad Prism software v.7.0d. 

## 3. Results

### 3.1. Anthropometric, Metabolic, Lifestyle, and Health Status Evaluation

As previously reported, the KETO and MEDI groups did not differ significantly from each other at baseline, regarding anthropometric, metabolic, lifestyle, and health status variables. Both groups had a mean BMI value > 30 kg/m^2^, a mean waist circumference (WC) above the IDF reference values, and a mean HbA1c value > 6.5%. Additionally, they had a mean total cholesterol value > 200 mg/dL and a mean systolic BP value of approximately 130 mmHg. Regarding lifestyle, they showed an MDS (range 0–55) of 24.8 ± 7.8 for the KETO group and 26.8 ± 4.6 for the MEDI group and a PAL close to the sedentary level (700 METs/week). Finally, the SF-36 PCS and MCS mean scores (range 0–100) were between 40 and 50 in both groups [51].

At T3, the KETO group showed a more significant reduction in body weight, BMI, WC, and FM% compared to the MEDI group. However, there was only a slight but non-significant difference between the groups regarding metabolic variables. Concerning lifestyle, both groups showed a reduction in daily caloric intake and an increase in adherence to the MD, while the PAL and daily sitting time remained unchanged. In the KETO group, a significant improvement in both the PCS and MCS scores of the SF-36 was observed, while the MEDI group showed a slight worsening, indicated by a reduction in both scores [51]. At T6, as shown in Supplementary Table S1, the KETO group had a further slight reduction in anthropometric indices (body weight, BMI, WC, and FM%) compared to T3. The MEDI group showed a significant reduction in BMI (*p* = 0.0354) and FM% (*p* = 0.0295). As for FFM, it remained stable in both groups. Regarding the variations in metabolic variables between T3 and T6, the KETO group showed a non-significant increase in the mean values of FPG, HbA1c, LDL cholesterol, and TG and a significant increase in the mean value of total cholesterol (*p* = 0.0331). A non-significant decrease in the mean values of all the metabolic variables was observed in the MEDI group.

The lifestyle evaluation highlighted in both groups a slight, non-significant improvement in adherence to the MD and PAL, as well as in the physical aspects of health status (SF-36 PCS). On the other hand, the mental aspect index (SF-36 MCS) showed a slight decrease in the KETO group and a slight increase in the MEDI group. Regarding the total caloric intake, a slight increase and a slight decrease were observed in the KETO group and the MEDI group, respectively.

The comparison of the difference in mean values between T3 and T6 for each variable in both groups did not yield any significant results (see Supplementary Material Table S2 online).

When we consider the comparison between T0 and T6, both groups showed a significant reduction in body weight, BMI, WC, and FM% mean values. However, in the KETO group, a significant reduction in FFM was observed. As for metabolic variables, both groups showed a reduction in FPG, HbA1c, total cholesterol, and TG mean values, as well as an increase in HDL cholesterol mean values. The reduction reached statistical significance exclusively for HbA1c (*p* = 0.0201) and TG (*p* = 0.0484) mean values in the KETO group. Regarding the other variables, the comparison between T0 and T6 highlighted a reduction in the caloric intake, an improvement in adherence to the MD and PAL in both groups, and a significant improvement of the PCS in the KETO group. As for MCS, the increase and the decrease in the mean values, respectively, observed in the KETO and MEDI groups were not statistically significant. These results are shown in Table 1.

Furthermore, comparing the difference in mean values obtained at T0 and T6 for each variable in both groups, the KETO group showed a more significant reduction in body weight, BMI, WC, FFM, and a significant improvement in PCS (see Supplementary Table S3 online).

The T12 evaluation showed a worsening of several parameters, when compared to the T6 evaluation. Specifically, the KETO group showed a slight increase in body weight and BMI and a significant increase in WC (*p* = 0.0324), FM% (*p* = 0.0079), total and LDL cholesterol (*p* = 0.0437; *p* = 0.0295), and DBP (*p* = 0.0305) values, while in the MEDI group, a significant increase in total cholesterol levels (*p* = 0.0437) and a significant decrease in SF-36 PCS. (*p* = 0.0377) were observed. However, an increase in the phase angle and in HDL cholesterol mean values in both groups was also observed, which was significant in the MEDI group (*p* = 0.0246; *p* = 0.0031) (see Table 2).

The comparison of differences between the two groups revealed a more significant increase in HDL cholesterol mean value in the MEDI group than in the KETO group (see Supplementary Table S4 online). Finally, considering the comparison between the baseline and the final evaluations, a reduction in the mean values of body weight, BMI, WC, and FM%, as well as a reduction in FPG, HbA1c, and TG mean values, was observed in both groups. All these variations were not significant, except for BMI in the MEDI group (*p* = 0.0445).

Additionally, both groups had an increase in the mean values of phase angle and an improvement in the lifestyle variables. However, some variables showed a different behavior between the two groups. More specifically, FFM showed a decrease in the KETO group, while a significant increase was observed in the MEDI group (*p* = 0.0416). Moreover, a significant increase in DBP mean values (*p* = 0.0377) and a significant reduction in energy intake (*p* = 0.0493) were observed in the KETO group. Furthermore, the SF-36 questionnaire analysis highlighted an improvement in the KETO group and a worsening, statistically significant MCS in the MEDI group (*p* = 0.0102) (see Table 3).

The comparison of the differences in the mean values of the analyzed variables between the two groups from the baseline to the end of the observation period revealed a significant result for the variation in FFM, which increased in the MEDI group and decreased in the KETO group, and for the SBP mean value, increased in the KETO group and decreased in the MEDI group. Another statistically significant result was seen in the variation in the MCS mean value, which decreased in the MEDI group and increased in the KETO group (see Supplementary Materials Table S5 online).

### 3.2. Gut Microbiota Analysis

The sequencing depth showed excellent coverage (>99.993% for all samples); samples were normalized to 22,173 reads (the lowest number of reads per sample), as previously described [51].

#### 3.2.1. Alpha and Beta Diversity Analyses

**Figure 1:**
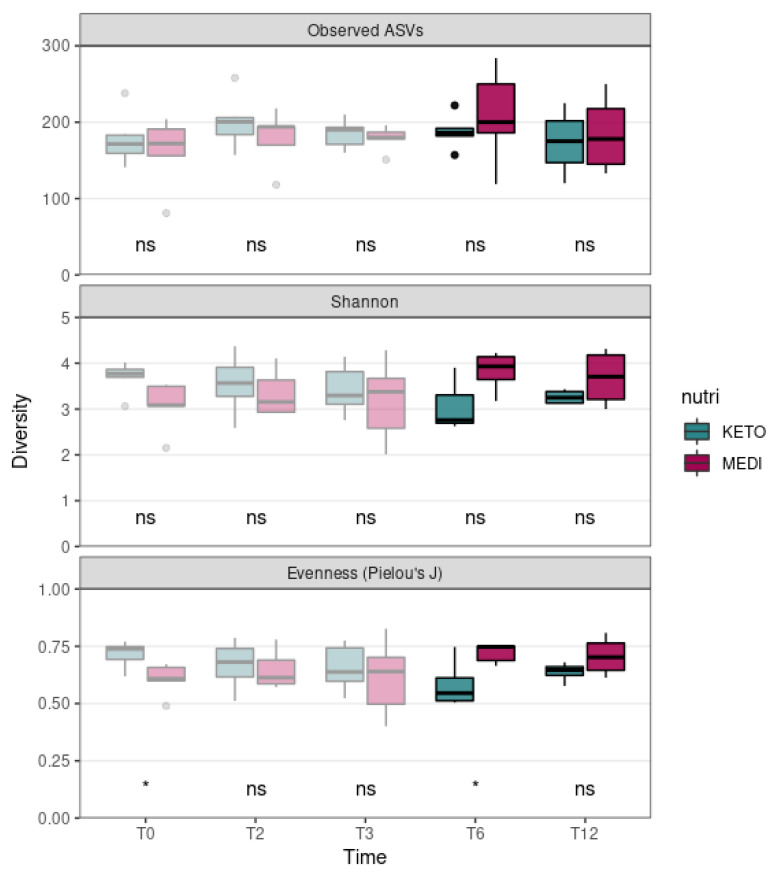
Gut microbiota alpha diversity comparison between diets at each time point. Each subplot concerns a different alpha diversity metric (observed ASVs, Shannon, or Pielou’s J) analyzed at baseline (T0), after two months (T2), after three months (T3), after six months (T6), and after twelve months (T12) of nutritional intervention. The boxes highlight the median value and cover the 25th and 75th percentiles, with whiskers extending to the more extreme values that are within 1.5 times the length of the box. Statistical significance was evaluated using the Mann–Whitney U test with significance levels indicated as follows: ns (non-significant); * (*p* ≤ 0.05). *p*-values are shown in Supplementary Table S6 online. KETO (in green) = patients who have sequentially followed a very-low-calorie ketogenic diet and a Mediterranean diet (VLCKD-MD); MEDI (in purple) = patients who followed a low-calorie Mediterranean diet (MD). Results up to T3 follow-up (already published [51]) are shown in pale colors, while results up to T12 are shown in bright colors.

We evaluated the differences in alpha diversity across the two nutritional groups by extending the follow-up analysis relating to T3 (pale colored plot, Figure 1) up to the twelfth month (T12) of nutritional intervention (bright colored plot, Figure 1). In particular, the Mann–Whitney U test showed no statistically significant differences in the ASV richness and in the Shannon index at any time point, while the Pielou’s J index, which was already significantly higher at baseline in the KETO group compared to the MEDI group (*p* = 0.017), appeared to be significantly reduced at T6 in the KETO group compared to the MEDI group (*p* = 0.032, see Figure 1 and Supplementary Table S6 online).

**Figure 2:**
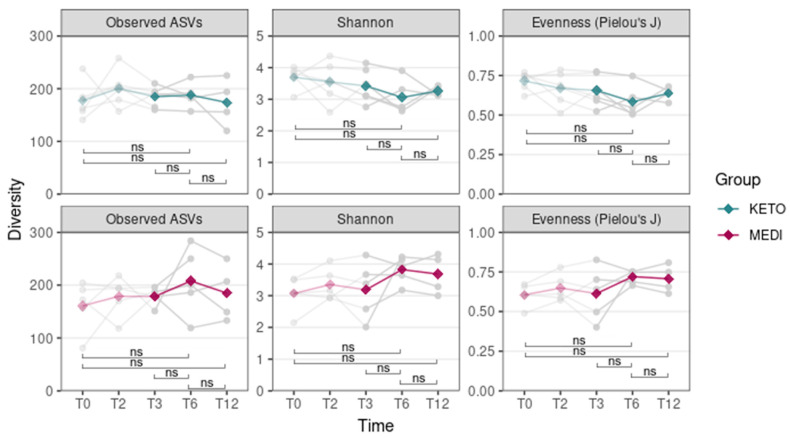
Gut microbiota alpha diversity comparison between time points for each diet. Each subplot concerns a different combination of alpha diversity metric (observed ASVs, Shannon, or Pielou’s J) and diet (KETO or MEDI). The green (KETO group) and purple (MEDI group) lines refer to the median value of indexes at each time point. In the background, each patient’s index value at each time point is represented in light gray. Statistical significance was evaluated using the paired Wilcoxon signed-rank test with significance levels indicated as follows: ns (non-significant). *p* equal to or less than 0.05 was considered statistically significant. KETO = patients who followed a very-low-calorie ketogenic diet (VLCKD); MEDI = patients who followed a low-calorie Mediterranean diet (MD); pale-colored plot = results up to T3 follow-up that are already published [51]; bright-colored plot = new results relating to the analyzes extended up to T12. Samples were analyzed at baseline (T0), after two months (T2), after three months (T3), after six months (T6), and after twelve months (T12) of nutritional intervention.

Moreover, no statistically significant differences in all alpha diversity indices (assessed by performing a Wilcoxon paired test between different time points) throughout the duration of NI in the KETO and MEDI groups were observed (Figure 2 and Supplementary Tables S7 and S8 online).

Concerning the beta diversity, the compositional trajectory of each patient along the nutritional intervention is shown in Supplementary Figure S1 online.

**Figure 3:**
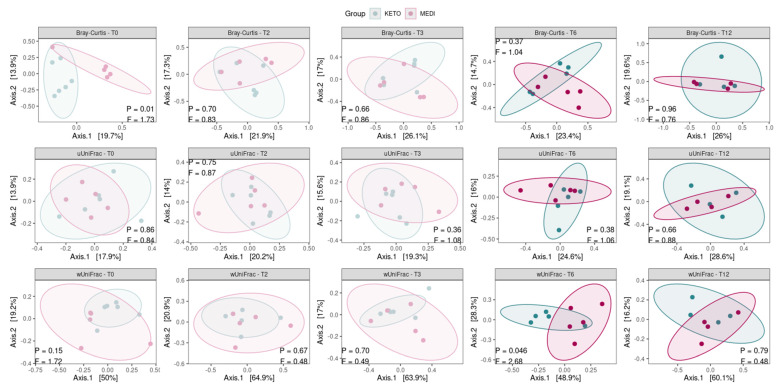
Principal coordinate analysis plots with comparisons of gut microbiota composition between diets at each time point. Each subplot concerns a combination of beta diversity metric (Bray–Curtis, unweighted UniFrac, or weighted UniFrac) and time points (T0, T2, T3, T6, and T12). Ellipsoids depict the 90% compositional confidence interval. Statistical significance was evaluated using the PERMANOVA test, with statistical summaries included in each subplot. *p* equal to or less than 0.05 was considered statistically significant. KETO = patients who have sequentially followed a very-low-calorie ketogenic diet and a Mediterranean diet (VLCKD-MD), MEDI = patients who followed a low-calorie Mediterranean diet (MD).

The principal coordinates analysis (PCoA) based on the weighted UniFrac matrix showed a marked separation between the GM communities of the KETO and MEDI groups at T6 (see Figure 3 and Supplementary Table S9 online), confirmed by PERMANOVA analysis, which indicated a significant difference in beta diversity between cohorts (sum of squares = 0.23, F = 2.68, R^2^ = 0.25, *p* = 0.046). However, the beta diversity based on the weighted UniFrac matrix at T12, unweighted UniFrac, and Bray–Curtis dissimilarity metrics across the two nutritional groups did not show any statistically significant difference, as shown in Figure 3 and in Supplementary Table S9 online.

**Figure 4:**
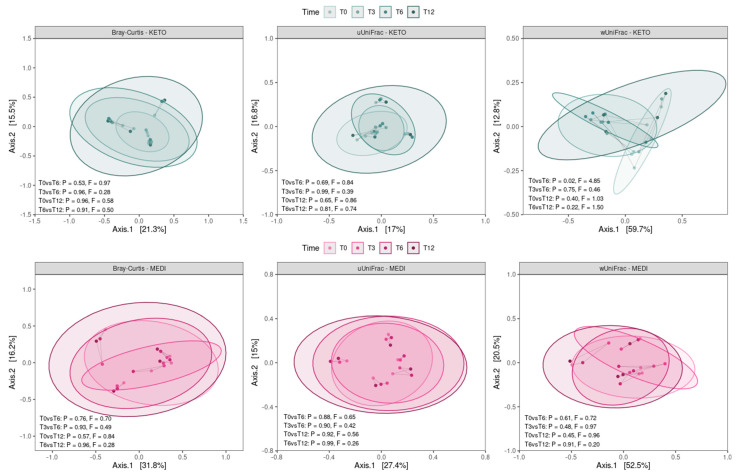
Principal coordinate analysis plots with comparisons of gut microbiota composition between time points for each diet. Each subplot concerns a combination of beta diversity metric (Bray–Curtis, unweighted UniFrac, or weighted UniFrac) and diet (KETO or MEDI). Ellipsoids depict the 90% compositional confidence interval. Samples from the same patient are linked by a gray line. Statistical significance was evaluated using the PERMANOVA test, with statistical summaries included in each subplot. *p* equal to or less than 0.05 was considered statistically significant. KETO = patients who have sequentially followed a very-low-calorie ketogenic diet and a Mediterranean diet (VLCKD-MD), MEDI = patients who followed a low-calorie Mediterranean diet (MD). Samples were analyzed at baseline (T0), after two months (T2), after three months (T3), after six months (T6), and after twelve months (T12) of nutritional intervention.

By performing a pairwise analysis between each time point throughout each nutritional intervention, a significant difference in beta diversity was obtained exclusively for the weighted UniFrac matrix in KETO, which showed a marked separation between the GM communities at T0 vs. T6, confirmed by PERMANOVA analysis, which indicated a significant difference in beta diversity between the cohorts (see Figure 4 and Supplementary Tables S10 and S11 online).

#### 3.2.2. Compositional Analysis of Intestinal Microbiota

No statistically significant differences in the Firmicutes/Bacteroidota ratio between time points in both diet groups were detected (see Supplementary Figure S2 and Tables S12 and S13 online).

Regarding the taxonomic analysis for each study cohort, we first compared the relative abundance of each taxon at baseline with that achieved after the initiation of each NI without considering each time point (with repeated sampling from the same individuals). The results, see Supplementary Figure S3 online, showed different statistical significances in the KETO cohort, while only three microbial taxa were significantly altered in the MEDI cohort. The first substantial difference between the two study cohorts was the opposite trend of quantitative changes in the Verrucomicrobiota phylum, for which a significant reduction in the MEDI group after the onset of MD was observed. Both cohorts were in line with a significant increase in the Christensenellales order and the Christensenellaceae family.

To evaluate the taxonomic changes longitudinally for both diets, we carried out a Generalized Linear Mixed-effects Model analysis by comparing the data for each time point (T6 and T12) prospectively and with baseline (T0).

**Figure 5:**
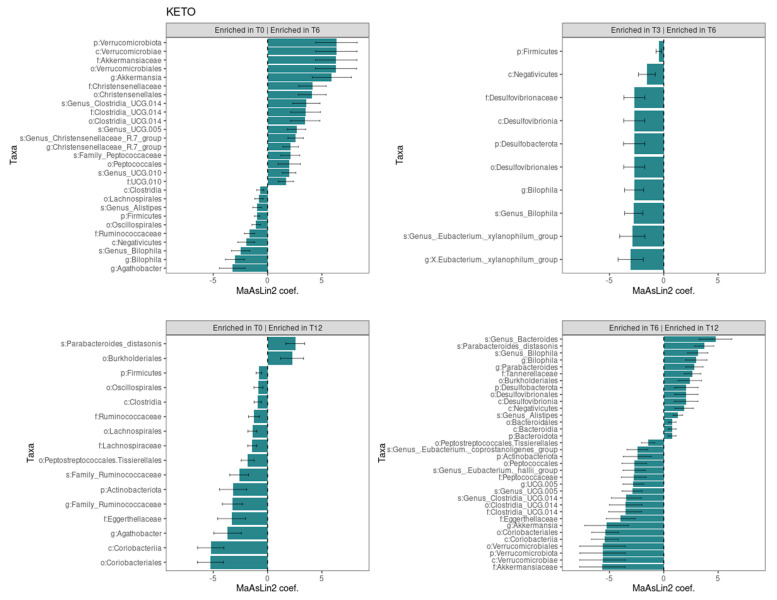
Significant changes in gut microbiota taxa abundance between time points in KETO group. Each subplot concerns a comparison between time points (T0 vs T6, T0 vs T12, T3 vs T6, and T6 vs T12) in the KETO group. Statistical significance was evaluated by running a Generalized Linear Mixed-effects Model with MaAsLin2. Effect size is represented by the MaAsLin2 model coefficients and respective standard errors. Only taxa abundance changes at *p* ≤ 0.05 and *q* ≤ 0.25 are considered statistically significant. q: p adjusted with the Benjamini–Hochberg (BH) correction test with a cut-off at *q* ≤ 0.25. KETO = patients who have sequentially followed a very-low-calorie ketogenic diet and a Mediterranean diet (VLCKD-MD). Samples were analyzed at baseline (T0) and after two (T2), three (T3) (see previously published data from the short follow-up [51]), six (T6), and twelve (T12) months of nutritional intervention. No significant data were observed at T6 and T12 in the MEDI group.

The Generalized Linear Mixed-effects Model, confirmed after multiple testing corrections with a cut-off at *q* ≤ 0.25, showed several significant microbial markers associated with the ketogenic diet over time, while the Mediterranean diet showed no significant impact on the taxonomic profile (see Figure 5). Results were ranked by their MaAsLin2 coefficient: the Verrucomicrobiota phylum was identified as the main biomarker in KETO, together with its members Verrucomicrobiae, Akkermansiaceae, Verrucomicrobiales, and *Akkermansia* at T6 compared with baseline, while the comparison of T3 with T6 of the same taxa showed no significant changes. Within the Firmicutes phylum, the strongest associations were also related to the Christensenellaceae family, the Christensenellales order, and, to a lesser extent, to the *Christensenellaceae R7 group* genus and *Genus Christensenellaceae R7 group* species at T6 compared with baseline. Other bacterial members belonging to the Clostridia UCG-014 order, to the UCG-010 family, and to the Peptococcales order were elevated at T6 compared with baseline. After six months of nutritional intervention, an opposite trend to what we previously observed for the members belonging to the Verrucomicrobiota phylum was highlighted; the *Akkermansia* genus, together with the Akkermansiaceae family, the Verrucomicrobiales order, the Verrucomicrobiae class, and the Verrucomicrobiota phylum, was significantly reduced and showed the greatest value of MaAsLin2 coefficient. A similar trend was observed within the Firmicutes phylum for the unclassified Clostridia UCG-014 order, family, and species; for the *Genus UCG-005* species; for the Peptococcales order; and for the Peptococcaceae family. A strong association was also found for the increase in the unclassified *Genus Bacteroides* species and in the *Parabacteroides distasonis* species at T12 compared with T6.

In addition, we classified the longitudinal trend, from baseline to T12, of all significances in the KETO group (see Supplementary Table S14 online) in relation to the previous results obtained in the short-term follow-up [51].

Some of the significant data observed in the KETO group were graphically represented at the phylum and genus levels for both the KETO and MEDI groups (see Supplementary Figure S4).

#### 3.2.3. Spearman Correlation Between Gut Microbiota Alterations and Clinical Variables at Baseline

**Figure 6:**
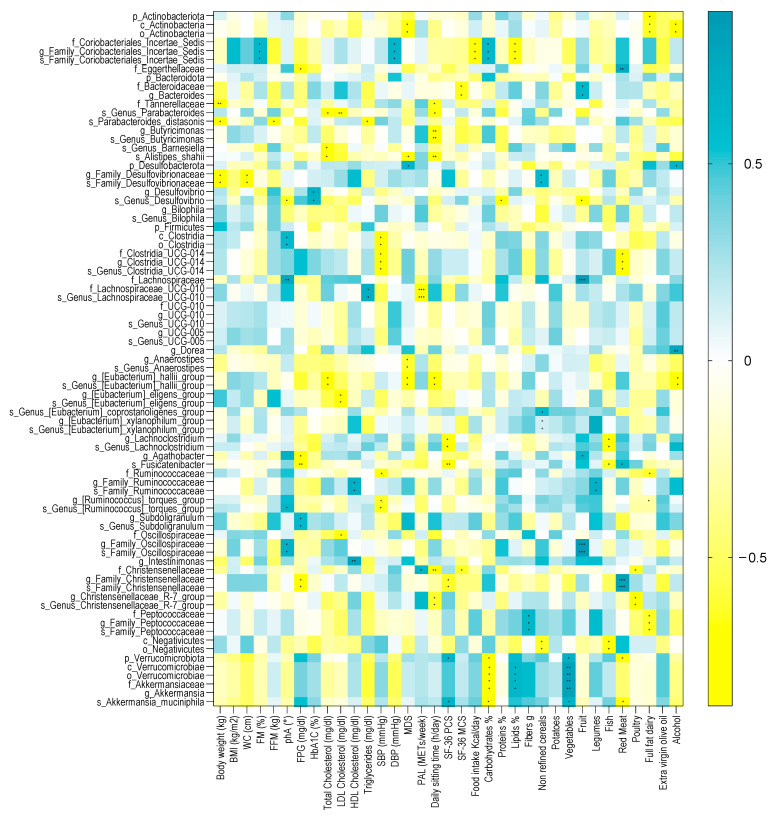
Spearman correlation analysis between significant GM alterations and clinical and nutritional variables in patients with diabesity at baseline. Only significant bacterial taxa identified in the Generalized Linear Mixed-effects Model were correlated to clinical and nutritional parameters. The correlation heatmap is used to represent significant statistical correlation values (Rho) between significant GM alterations and clinical and nutritional variables in patients with diabesity at baseline; blue squares indicate significant positive correlations (Rho > 0.5, *p* ≤ 0.05) and yellow squares indicate significant negative correlations (Rho < −0.5, *p* ≤ 0.05). *p*-value equal to or less than 0.05 was considered statistically significant. * = *p* ≤ 0.05; ** = *p* ≤ 0.01; *** = *p* ≤ 0.001. BMI = body mass index, WC = waist circumference, FM (%) = fat mass expressed in percentage, FFM = free fat mass expressed in kilograms, phA (°) = phase angle, FPG = fasting plasma glucose, HbA1c = glycosylated hemoglobin, SBP = systolic blood pressure, DBP = diastolic blood pressure, MDS = Mediterranean diet score, PAL = physical activity level, METs/week = metabolic equivalent of task-minutes per week, SF-36 PCS = physical component summary of SF-36, and SF-36 MCS = mental component summary of SF-36. Spearman’s correlations were calculated in GraphPad Prism software v.7.0d.

Several microbial taxa, found to be significant with the Generalized Linear Mixed-effects Model, showed a significant correlation with clinical and nutritional parameters in patients with T2DM at baseline. A heatmap correlation is shown in Figure 6, while the coefficient values and *p* values for each significant correlation can be found in Supplementary Table S15 online.

Among the anthropometric measurements, body weight was inversely correlated with the unclassified *Family Desulfovibrionaceae* genus and species, with the Tannerellaceae family, and with the *P. distasonis* species. Within the Desulfobacterota phylum, the unclassified *Family Desulfovibrionaceae* genus and species were inversely correlated with WC. The *P. distasonis* species was also negatively correlated with FFM. Furthermore, the unclassified Coriobacteriales incertae sedis family and the unclassified *Family Coriobacteriales incertae sedis* genus and species were positively correlated with FM.

Among the metabolic parameters, phA was positively correlated with the Clostridia class, the unclassified Clostridia order, the Lachnospiraceae family, the unclassified *Family Oscillospiraceae* genus and species, and with the unclassified *Genus Ruminococcus torques group* species. Furthermore, the unclassified *Genus Desulfovibrio species* was inversely correlated with phA. FPG was also inversely correlated with *Fusinicatenibacter*, *Agathobacter*, the unclassified *Family Christensenellaceae* genus and species, and the Eggerthellaceae family and was positively correlated with the *Subdoligranulum* genus and the unclassified *Subdoligranulum* species. HbA1C was positively correlated with *Desulfovibrio* and the unclassified *Genus Desulfovibrio* species.

Concerning the lifestyle factors, the PAL score was positively correlated with the Christensenellaceae family and negatively correlated with the Lachnospiraceae UCG-010 family and species, while the daily sitting time inversely correlated with the Christensenellaceae family, the *Christensenellaceae R7 group* genus and species, the *E. hallii group* genus and species (Firmicutes phylum), with Tannerellaceae, the unclassified *Genus Parabacteroides* species, the *Butyricimonas* genus, the unclassified *Genus Butyricimonas* species, and *Alistipes shahii*. MDS negatively correlated with the *E. hallii group* genus and species, the *Anaerostipes*, the unclassified *Genus Anaerostipes* species, *Alistipes shahii*, the Actinobacteria class, and the unclassified Actinobacteria order, while positively correlated with the Desulfobacterota phylum.

The nutritional variables significantly correlated with several microbial taxa. Among those who showed a strong association coefficient over time with the VLCKD, we found that the Verrucomicrobiota phylum, the Verrucomicrobiae class, the Verrucomicrobiae order, the Akkermansiaceae family, the *Akkermansia* genus, and *Akkermansia muciniphila* were negatively correlated with carbohydrate intake and positively correlated with vegetable intake. Verrucomicrobiota and *Akkermansia muciniphila* were negatively correlated with fish intake, while the Verrucomicrobiae class, the Verrucomicrobiae order, the Akkermansiaceae family, and the *Akkermansia* genus were positively correlated with lipids intake. Moreover, Verrucomicrobiota and *Akkermansia muciniphila* were positively correlated with SF-36 PCS. The *Family Christensenellaceae* genus and species positively correlated with red meat intake, while the Christensenellaceae family and the *Christensenellaceae R7 group* genus and species were negatively correlated with poultry intake.

#### 3.2.4. Functional Metagenome Prediction Analysis

**Figure 7:**
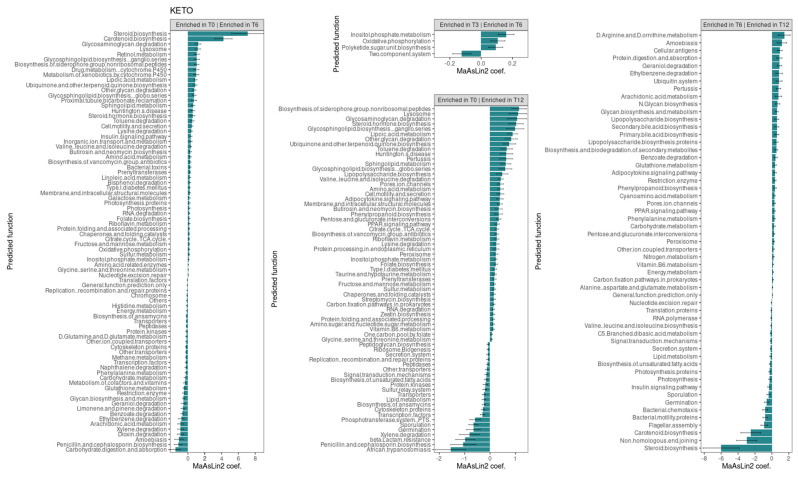
Changes in the abundance of gut microbiota predictive functions between time points in the KETO group. Each subplot concerns a comparison between time points (T0 vs. T6, T0 vs. T12, T3 vs. T6, and T6 vs. T12) of the abundance of gut microbiota predictive functions in the KETO group. Statistical significance was evaluated by running a Generalized Linear Mixed-effects Model with MaAsLin2. Effect size of the association is represented by the MaAsLin2 model coefficients and respective standard errors. Only predicted function abundance changes at *p* ≤ 0.05 and *q* ≤ 0.25 are considered statistically significant. q: p adjusted with the Benjamini–Hochberg (BH) correction test with a cut-off at *q* ≤ 0.25. KETO = patients who have sequentially followed a very-low-calorie ketogenic diet and a Mediterranean diet (VLCKD-MD). Samples were analyzed at baseline (T0) and after two (T2), three (T3) (see previously published data from the short follow-up [51]), six (T6), and twelve (T12) months of nutritional intervention. No significant data were observed at T6 and T12 in the MEDI group.

Consistent with the taxonomic analysis, we found several metabolic pathways that changed significantly over time only in the KETO group (see Figure 7). Specifically, the strongest associations were positively related to steroid and carotenoid biosynthesis in T6 compared to baseline (their significant increase was already observed at both T2 and T3 in comparison to baseline [51]), while they were significantly reduced after the sixth month of NI until T12. Furthermore, even the non-homologous end-joining pathway, which was increased at both T2 and T3, was significantly reduced from T6 to T12.

At T6, compared to baseline, an association was also observed, albeit to a lesser extent, for the pathways involving glycosaminoglycan degradation, lysosome, retinol metabolism, glycosphingolipid biosynthesis ganglion series, the biosynthesis of siderophore group non-ribosomal peptides, the drug metabolism of cytochrome P450, the metabolism of xenobiotics by cytochrome P450, lipoic acid metabolism, ubiquinone and other terpenoid quinone biosynthesis, other glycan degradation, and glycosphingolipid biosynthesis globo-series. All these pathways, except for the retinol metabolism, the drug metabolism of cytochrome P450, and the metabolism of xenobiotics by cytochrome P450 pathways, were also significantly increased at T12 compared to baseline. Furthermore, all these pathways, except for glycosphingolipid biosynthesis globo-series, were significantly increased at T2, while lysosome, retinol metabolism, the biosynthesis of siderophore group non-ribosomal peptides, and lipoic acid metabolism were also increased at T3.

Valine, leucine, and isoleucine degradation as well as the insulin signaling pathway were increased at both T6 and T12 compared with baseline (as they were present at both T2 and T3 [51]) and significantly reduced after the sixth month of NI until T12.

Several pathways were significantly reduced overtime. Specifically, energy metabolism, other transporter, histidine metabolism, peptidases, D-glutamine and D-glutamate metabolism, methane metabolism, cytoskeleton proteins, naphthalene degradation, restriction enzyme, ethylbenzene, limonene and pinene degradation, penicillin and cephalosporin biosynthesis, and dioxin degradation were significantly reduced at T6 (for which we had already observed their significant reduction at both T2 and T3 [51]). The glycan biosynthesis and metabolism, geraniol degradation, and carbohydrate digestion and absorptions pathways were similarly reduced at T6 (they were also significantly reduced at T3). Instead, the transcription factors, benzoate degradation, and xylene degradation pathways, which were significantly reduced at T6, were also significantly reduced at T2. Among these pathways significantly reduced at T6, energy metabolism, restriction enzyme, glycan biosynthesis and metabolism, geraniol degradation, benzoate degradation, and ethylbenzene increased significantly after the sixth month of NI until T12.

In addition, the pathways involving glutathione metabolism, amoebiasis, arachidonic acid metabolism, and carbohydrate metabolism were significantly reduced only at T6 (for which no significant alterations were found at T2 and T3), and they increased significantly after T6 compared to T12.

The phenylalanine metabolism, which was reduced at both T3 compared to T2 and at T3 compared to baseline, was similarly significantly reduced at T6 compared to T0, but it significantly increased after the sixth month of NI.

## 4. Discussion

The present prospective study is aimed at evaluating the mid-term (up to six months) and long-term (up to 12 months) impact of the ketogenic (KETO group) and Mediterranean (MEDI group) dietary patterns on the intestinal microbiota and on the anthropometric, clinical, and metabolic parameters of patients with obesity or overweight, who have recently been diagnosed with T2DM. This study extends previously published results [51] that evaluated the short-term (three months) impact on the same patients.

At baseline, patients were randomly instructed to follow either a VLCKD or an MD. At the end of the second month, as outlined in the study design [51], the group initially assigned to the VLCKD was re-evaluated and gradually switched to the MD (VLCKD-MD).

Consequently, from the fourth month onward, both groups followed a similar dietary protocol for the remaining observation period.

For the mid-term and long-term follow-ups, the samples were collected and analyzed after six (T6) and twelve months (T12) prospectively, and then compared with baseline (T0). Concerning the GM, the analysis was also performed by comparing samples before and after the initiation of the NI without considering each time point.

Regarding the anthropometric evaluation, our results showed that at T6, both groups had a significant reduction in body weight, BMI, WC, and FM compared to baseline. However, when we compared the differences as delta values for these variables between the two observation points, the reduction was more pronounced in the KETO group than in the MEDI group, with a statistically significant result for body weight, BMI, and WC. This finding is primarily attributable to the more significant weight loss achieved in the KETO group during the VLCKD phase in the first three months of the study [51], followed by only a slight further reduction in the subsequent three months. The marked weight loss in the KETO group, particularly during the ketosis phase, could also explain the significant reduction in FFM observed in this group between T0 and T3, followed by a stabilization of FFM between T3 and T6 when the KETO group switched from the VLCKD to the MD. Indeed, this finding is commonly observed during significant weight loss, even after following a VLCKD, and it may be considered a risk factor for weight regain despite the paucity of long-term data. On the other hand, in the MEDI group, as expected with the moderately hypocaloric dietary protocol, weight loss was more gradual, reaching statistical significance only in the mid-term.

The metabolic results revealed that, although both groups showed a reduction, only the KETO group experienced a significant decrease in mean HbA1c and TG levels. This finding is supported by a recent meta-analysis of twenty-nine studies which observed significant improvements in glycemic and lipid profiles among patients with T2DM who adhered to a VLCKD protocol, suggesting a potential cardioprotective role for this nutritional approach [63].

Similar results were found in a previous meta-analysis of thirteen studies conducted on patients with T2DM, which reported a beneficial effect of the ketogenic diet on glycemic control. This dietary approach led to an average HbA1c reduction of 1.07%, along with improvements in insulin resistance, lipid profile, and obesity levels [64]. Given the close relationship between central and visceral obesity and insulin resistance, the more pronounced weight loss and reduction in waist circumference observed in our KETO group, compared to the MEDI group, may explain the more significant metabolic improvements seen in KETO patients. Finally, in our study, both groups showed an improved lifestyle, particularly in adherence to the MD which resulted in a higher mean score compared to both the baseline and T3 evaluations, but it was more pronounced in the MEDI group, specifically between the baseline and T6 evaluations. The increased attention to eating habits and the regular clinical and nutritional follow-up likely played a key role in this outcome, as well as in the reduction in total daily energy intake observed in both groups. On the other hand, despite a slight increase in the physical activity level at the sixth month mark, both groups remained predominantly sedentary. It should be noted that physical activity was assessed using the short form of IPAQ. While this is a standardized and widely used tool, it may not provide the same level of accuracy as an objective measurement, which could be considered a limitation. However, this outcome may be explained by the study’s primary focus on nutritional intervention, although supplemented with general advice on the positive effects of an active lifestyle on health. It should also be emphasized that this study was conducted during the COVID-19 pandemic, and the restrictions on people’s movements may have played a role in the results.

Regarding the perception of health status compared to baseline, the difference between the two groups was evident in both the physical and the mental aspects of quality of life, which improved in the KETO group but worsened in the MEDI group. Specifically, only the KETO group showed a significant improvement in the physical aspect of quality of life, consistent with the results observed at the third month of treatment. These results are linked to the significant improvements observed in the KETO group during the first three months of nutritional treatment, which may be attributed to the positive impact of carbohydrate restriction on mood [65]. Moreover, the rapid and significant weight loss, along with the reduced sense of hunger promoted by the VLCKD [66], played a fundamental role in enhancing the well-being of KETO patients. Indeed, during the subsequent three months, marked by the change in dietary protocol and weight regain, the KETO group experienced a slight decline in MCS value. On the other hand, the MEDI group showed a slight improvement in MCS value between T3 and T6, likely linked to the weight loss achieved during this period.

Our findings are partially consistent with those of Moriconi et al., who observed a significant weight loss and glucometabolic improvement in patients with T2DM on a VLCKD after three months of nutritional treatment. In contrast, patients following a Mediterranean, low-calorie diet did not show any significant change. Additionally, the same study reported a significant improvement in quality of life, measured by the SF-36 questionnaire, but only in the VLCKD group [67].

In contrast to these findings, our study showed a less pronounced difference between the two groups, highlighting that the MEDI group also achieved significant weight loss after the sixth month of nutritional treatment. At the same time point, compared to baseline, we observed an almost significant reduction in total daily energy intake and a marked improvement in adherence to the Mediterranean diet in the MEDI group, which may indicate good compliance with the dietary protocol. Additionally, in the MEDI group, we observed an improvement, although not significant, in both the physical and mental aspects of quality of life, likely attributable to the satisfaction and overall well-being associated with weight loss.

In the study by Moriconi et al. [67], the improvements observed after three months of the VLCKD protocol were maintained at twelve months. This led to a reduction in the need for T2DM medications in the VLCKD group and, in some cases, resulted in disease remission [67].

In our study, the twelve-month follow-up showed an overall decrease in body weight, BMI, WC, and FM compared to baseline in both groups. Specifically, BMI decreased significantly in the MEDI group. However, when comparing the results at twelve months with those at six months, there was a trend towards weight regain in both groups, with the trend being more pronounced, although not statistically significant, in the KETO group compared to the MEDI group. Specifically, the KETO group showed a statistically significant increase in waist circumference and fat mass. A possible explanation for this result is the more pronounced response of visceral fat to the progressive increase in carbohydrate intake in the KETO group. Moreover, it could be hypothesized that after the VLCKD phase, patients in the KETO group might have been less compliant with the dietary protocol.

Regarding body composition, an interesting finding was seen in the stable fat free mass and the increase in phase angle observed in both groups, which was significant in the MEDI group. This suggests improved cell quality, particularly in muscle cells. Another positive result is the maintenance of good glucometabolic status up to T12 in both groups. Indeed, none of them required pharmacological treatment for T2DM. This result is consistent with findings from other studies; specifically, a meta-analysis of forty-two randomized controlled trials conducted on patients with T2DM compared ten different diets and reported significant effects on glycemic control with both the ketogenic and Mediterranean diets [68]. Additionally, regarding the lipid profile in our study, both groups experienced an increase in HDL cholesterol levels and a decrease in triglyceride levels. On the other hand, as observed in Moriconi’s study [67], the two groups differed in their perception of health status, which improved in the KETO group but worsened in the MEDI group from baseline to the end of the study. Particularly, the improvement in the physical component of health status in the KETO group was more evident during the first period of the study, corresponding to the VLCKD phase, with a subsequent decline between T6 and T12.

Regarding the intestinal microbial composition, the abundance of taxa was significantly more homogeneously distributed at T6 in the MEDI group compared to the KETO group, whereas at T12, the differences in distribution homogeneity between the two study groups were no longer observed. On the contrary, the evenness was significantly higher in the KETO group compared to the MEDI group at baseline, as previously reported [51]. To assess the extent of this change, we performed additional analyses to investigate changes in alpha diversity over time for each study group, as well as in beta diversity (both between study groups and within each study group over time). Specifically, no significant changes in alpha diversity over time in both the KETO and MEDI groups were observed. Significant changes in beta diversity indices emerged only after the sixth month (T6) of nutritional intervention in the KETO group, while no significant differences during the short-term, as already described [51], and long-term (T12) treatments were observed. Therefore, at T6, the GM of the KETO group would appear to be characterized by certain very-abundant taxa. At T12, both study cohorts exhibited an opposite, though non-significant, trend, with respect to all alpha diversity indices.

Regarding the results from the beta diversity analysis, the two study groups differed significantly at the sixth month of nutritional intervention in a diversity-weighted measure that takes into account differences in the relative abundances of microbial taxa; no significant difference was observed neither in terms of qualitative diversity (considering only the presence or absence of taxa between the two cohorts) nor in the number of shared taxa and their relative abundance between the KETO and MEDI groups. The significant changes at T6 seem to result from more quantitative changes within the KETO group over time than from a significant taxonomic alteration at T6 within the MEDI group compared to baseline. In fact, by performing a prospective pairwise analysis between each time point during each type of NI, a significant difference in beta diversity was obtained exclusively for the KETO-weighted UniFrac matrix at T6 compared to baseline. These findings were confirmed in the taxonomic analysis, especially in the KETO group, in which greater and significant quantitative taxonomic changes compared to the MEDI group were observed, with significant variation over time.

Specifically, by assessing the quantitative taxonomic changes before and after the initiation of the nutritional intervention, with repeated sampling from the same individuals and without considering each follow-up time point, we observed different statistical significances in the KETO cohort, while only three microbial taxa were significantly altered in the MEDI cohort. The first substantial difference between the two study cohorts was the opposite trend in quantitative changes in the Verrucomicrobiota phylum, with a significant reduction in the MEDI group after the onset of the MD. Both cohorts exhibited a significant increase in the Christensenellales order and the Christensenellaceae family. Despite the decrease in the Verrucomicrobiota phylum to a greater extent at T6 and T12 compared with baseline in the MEDI group, the *p*-value was not statistical significance.

To longitudinally evaluate the taxonomic changes for both diets, we carried out a Generalized Linear Mixed-effects Model analysis by comparing the data for each time point (T6 and T12) prospectively and with baseline (T0). Additionally, we classified the longitudinal trend of all significant findings in the KETO group from baseline to T12 (see Supplementary Table S14 online) in relation to the previous short-term results [51]. In the present discussion, we analyzed the type of impact (beneficial or detrimental) of the VLCKD on GM, considering the pathophysiological implications of the different microbial taxa. Taxonomic changes over time and the pathophysiological implication of bacterial taxa are discussed in the following paragraphs.

We found that the VLCKD, in association with the MD (VLCKD-MD), had a more beneficial impact than the MD on the gut microbiota phenotype of patients with diabesity who were naïve to drug treatment, especially up to the sixth month (mid-term follow-up). In fact, several significant changes associated with the VLCKD-MD for each time point were observed, while no significant changes in the taxonomic profile were found in response to the Mediterranean diet.

To summarize, we conclude that the VLCKD-MD had a significant and progressive beneficial impact on the taxonomic profile from baseline to mid-term (T6) and long term (T12) follow-ups, as well as a limited beneficial impact on some bacterial taxa at certain time points, albeit not progressively. After the sixth month, most markers of human health decreased and were no longer significantly increased compared with baseline, though most of these taxa maintained non-significant abundance levels above baseline. Furthermore, after T6, both a positive and a negative modulatory effect of VLCKD-MD on changes in a few bacterial taxa were observed. Finally, the VLCKD-MD exercised a limited impact at specific time points during the short-term follow-up on some microbial taxa, though further investigation is required as the literature reports conflicting data on the role these taxa play in T2DM.

### 4.1. Progressive, Beneficial Effect of VLCKD-MD on Microbial Taxa from Baseline to Mid-Term (T6) and Long-Term (T12) Follow-Ups

#### 4.1.1. A Significant Increase over Time in Several Microbial Taxa up to T6

In the KETO cohort, we observed a beneficial effect of VLCKD-MD up to the sixth month of NI, attributed to the increase in bacterial taxa implicated in promoting intestinal health, glucose homeostasis, and insulin sensitivity and negatively associated with different inflammatory or metabolic diseases, as well as with the impairment of barrier function. It should be noted that the role of certain implicated bacterial taxa, such as the Peptococcaceae family, has yet to be fully defined. Quantitative changes in these bacterial taxa followed a mirror pattern before and after the sixth month of NI in the KETO group, with a significant increase at each time point up to T6 compared with baseline. It should be emphasized that after the sixth month, the beneficial impact of NI on the involved taxa was no longer observed due to their significant decrease up to T12. However, no harmful impact was observed either as most of these taxa have maintained abundance levels above baseline, although not significantly. Interestingly, the same trend is maintained between the lower and higher taxonomic levels. In particular, the Verrucomicrobiota phylum and its members (the Verrucomicrobiae class, the Verrucomicrobiales order, the Akkermansiaceae family, and the *Akkermansia* genus), the Clostridia UCG-014 order (and related Clostridia_UCG-014 family and genus and unclassified *Genus Clostridia UCG-014* species), the unclassified Genus UCG-005 species, and the Peptococcales order (with unclassified *Family Peptococcaceae* genus and species members and the Peptococcaceae family) followed the same trend (see Supplementary Table S14a online).

Furthermore, the Verrucomicrobiota phylum, together with its members mentioned above, was identified as the main biomarker in the KETO group. The role of *A. muciniphila* in promoting intestinal integrity, as well as intestinal and glucose homeostasis, and its effects on the improvement of metabolic parameters, both in cases of obesity and diabetes, are well documented [69,70,71,72,73,74,75]. Moreover, an increase in *A. muciniphila* has already been associated with KD [76], and its decrease was also considered a risk factor for T2DM [77,78]. In this study, as per Spearman’s analysis, all these taxa correlated significantly and negatively with carbohydrate intake and positively with vegetable intake at baseline. The Verrucomicrobiae class, the Akkermansiaceae family, and the *Akkermansia* genus correlate positively with lipid intake, while the Verrucomicrobiota phylum and the *A. muciniphila* species showed a significant increase with improved physical state perceived by patients and a significant reduction with the increase in red meat intake.

Other taxa that significantly increased up to T6 have also been associated with physiological homeostasis. As for Clostridia UCG-014, although a study evaluating the effect of berberine on hyperglycemia in T2DM rats showed a decrease [79], the administration of turmeric polysaccharides increased its abundance, together with that of *Lactobacillus*, both genera considered to be the main source of tryptophan metabolites, which regulate intestinal homeostasis [80,81,82]. In line with this, a reduction in Clostridia UCG-014 was associated with the impairment of barrier function in healthy relatives of patients with Crohn’s disease [83].

As for the *UCG-005* genus, it was found that the abundance of Oscillospiraceae UCG-005 was lower in patients with Crohn’s disease (CD) and ulcerative colitis (UC) than in healthy individuals [84].

Regarding the increase in Peptococacceae over time, it has been observed that fatty acids, as well as choline, produced also by this family, are involved in the regulation of glucose homeostasis, lipid metabolism, and insulin sensitivity [85,86]. In addition, several studies negatively associate members belonging to Peptococcaceae with disease status [87,88] or pre-disease status [89]. However, some data related to the Peptococcaceae family in the literature are in contrast [90,91], and those correlating this family to TMAO levels are conflicting [92,93,94]. In the present study, we found a positive and significant correlation between the Peptococcaceae family and fiber intake at baseline.

The positive modulatory effect of VLCKD-MD was also observed up to T6 due to the significant increase in some markers of human health and taxa negatively associated with disease and with the risk of T2DM. After T6, the abundance of these taxa did not change significantly, maintaining a stable relative abundance until the twelfth month of NI, although they did not increase significantly compared with baseline (see Supplementary Table S14b online). Specifically, the taxa included the Christensenellales order, the Christensenellaceae family, the unclassified Christensenellaceae R7 group genus and species, and the UCG-010 family, genus, and species. Christensenellaceae, similarly to the Verrucomicrobia phylum and its members, has been associated with human longevity [28,95,96,97,98], being a marker of human health. A reduction in Christensenellaceae was observed in individuals with pre-type 2 diabetes [86]. Moreover, the Christensenellaceae family has been associated with a lean phenotype and is negatively correlated with visceral fat mass and other related poor metabolic parameters [99,100]. These data are consistent with a significant reduction in body weight, BMI, WC, and FM in both the KETO and MEDI cohorts, as Christensenellales and Christensenellaceae increased significantly after the start of MD, not considering the different time points of follow-up, even in the MEDI group. The Christensenellaceae family has also been positively associated with healthy glucose metabolism [97,101]. Consistently, we found that the unclassified *Family Christensenellaceae* genus and species were negatively and significantly correlated with FPG and PCS at baseline.

The positive modulatory effect of VLCKD-MD was also observed with the increase in the unclassified UCG-010 family and its members. A recent study demonstrated a significant decrease in Oscillospirales UCG-010 in patients with colorectal cancer compared with healthy controls [102]. Moreover, Ruminococcaceae UCG-010 has been negatively associated with the risk of T2DM [88].

#### 4.1.2. A Significant Decrease over Time in Several Microbial Taxa up to T12

Further findings of this study corroborate the potential beneficial effect of the VLCKD-MD for its effect on the percentage reduction over time in bacterial taxa positively associated with body weight and fasting plasma glucose, negatively associated with free lean mass, and on bacterial taxa associated with diabesity, metabolic diseases, and obesity, which were reduced following antidiabetic treatments in T2DM models or following low-calorie diets and prebiotic supplementation (see Supplementary Table S14f online). Regardless, the role of certain implicated bacterial taxa, such as the Actinobacteriota phylum, has yet to be defined in T2DM.

Specifically, the Firmicutes phylum decreased significantly at all time points up to T12 compared to baseline. Other taxa belonging to the Firmicutes phylum showed reduced relative abundance compared with baseline, without maintaining statistical significance for each temporal comparison. Among them are the Clostridia class, the Oscillospirales, the Lachnospirales, the Peptostreptococcales, the Tissierellales orders, the Lachnospiraceae family (and the related *Agathobacter* genus), the Ruminococcaceae family, and the unclassified *Family Ruminococcaceae* genus and species.

Although we observed a significant reduction over time in Firmicutes in the KETO group, the Firmicutes/Bacteroidota ratio did not vary significantly. Notably, although the Bacteroidetes/Firmicutes ratio has previously been suggested as markers of metabolic disease, it has not shown consistent associations with T2DM [103].

The depletion in Firmicutes could be related to a reduction in body weight and an increase in lean body mass over time [27]. In the study by Ahmad A. and collaborators, Firmicutes and Actinobacteria correlated positively with fasting blood glucose, while Bacteroidetes and Proteobacteria were negatively correlated in patients with diabesity [104]. Fasting blood glucose was reduced in the KETO group, although not significantly, up to T12, and this agrees with a reduction over time in Actinobacteria and Firmicutes in the KETO group, but not with the change in Bacteroidota in the same cohort.

Regarding the lower taxonomic levels, the Clostridia class was found to be increased in subjects with diabesity, compared with healthy controls [104]; a remarkable increase in the abundance of the Clostridia class, higher levels of the Lachnospirales and Oscillospirales orders, a significant increase in the abundance of the Lachnospiraceae, Oscillospiraceae and Ruminococcaceae families, and a marked decrease in the Clostridia UCG-014 group in mice with diabetic nephropathy (DN) were also found [105]. Elevated levels of Peptostreptococcales-Tissierellales and Lachnospirales were found in T2DM rats following metformin treatment, compared with controls [106], while the antidiabetic effect of millet bran polysaccharides was associated not only with improvements in T2DM symptoms but also with a reduction in Firmicutes, Peptostreptococcales-Tissierellales, and Lachnospirales in an animal model of T2DM [106].

Despite the beneficial effects related to the *Oscillospira* genus, some studies have shown a strong positive association between *Oscillospira* abundance and several disorders [107], as well as with the development of diabetes in rats [108]. On the other hand, reduced abundances have been observed in T2DM rats [109].

The *Ruminococcus* genus, belonging to the Ruminococcaceae family, represents one of the taxa found to be associated with T2DM, and consistently, metformin therapy is associated with a reduction in Ruminococcaceae in T2DM [103]. In addition, a reduced abundance of the *Agathobacter* genus was found in patients with T2DM [110,111] and was associated with longer diabetes duration [110]. Furthermore, for its depletion, *Agathobacter* has been considered a promising biomarker of diabetic kidney disease (DKD) progression [112].

We also observed a reduction in the Actinobacteriota phylum over time, except for a slight increase above baseline at T6, albeit not significant. Consistently, other NIs based on a basic diet followed by KD on 17 adults, who were overweight or with class I obesity, without diabetes, showed a reduced relative abundance in Actinobacteria and Firmicutes, while the Bacteroidetes and Proteobacteria phyla increased [113]. In addition, an increase in the levels of Actinobacteria has been demonstrated in T2DM [114], in agreement with their positive correlation with fasting blood glucose [104], and in obesity [115]. On the contrary, its increase has been observed following sleeve gastrectomy (SG) in animal models, together with reduced blood glucose [116]. Furthermore, an increase in Actinobacteria concurrently with that of Firmicutes has been observed after T2DM treatment, and it was associated with better glycemic control or lipid profile at follow-up [117]. In this study, changes over time in Actinobacteriota could be partly related to the diet, as Actinobacteria encodes glycosyl hydrolases (GHs) and is involved in the biodegradation of resistant starch and plant-derived polysaccharides [118].

### 4.2. Limited Beneficial Effect of the VLCKD-MD on Microbial Taxa at Specific Time Points

#### 4.2.1. Significant Alterations in the Microbial Taxa Abundance in the Short-Term Follow-Up

We observed a positive effect of the VLCKD-MD up to T3 (see Supplementary Table S14c online) in the abundance of some microbial taxa associated with glucose homeostasis and insulin sensitivity [119,120,121], negatively associated with T2DM [122], as well as taxa negatively associated with fasting blood glucose (IFG) [123], which increased following polysaccharide treatment used to relieve T2DM [124]. The implicated taxa, including the *Eubacterium xylanophilum* group, the *Eubacterium eligens* group, and the *Intestinimonas* genus (all belonging to the Firmicutes phylum), increased up to the third month of NI in the KETO group (*Intestinimonas* increased up to T2), and then progressively decreased significantly at the sixth month and non-significantly at the twelfth month, in levels close to or above baseline. This suggests that the beneficial impact of the diet extends up to the third month of NI, while not proving detrimental in the longer follow-up.

A beneficial effect of VLCKD-MD in the short-term was found due to the reduction in the *Lachnoclostridium*, *Fusicatenibacter*, and *Ruminococcus torques* groups, as well as the *Dorea* genera (Firmicutes phylum) (see Supplementary Table S14c online). Interestingly, although *Lachnoclostridium*-harboring species produce SCFAs (mainly butyrate) with immuno-modulatory action [125,126], a reduction was observed in diabetes-induced cognitive impairment (DCI) models following tanshinone IIA treatment [127] and in mice with T2DM following the anti-hyperglycemic treatment [128]. In addition, its abundance has been associated with simple obesity [129], T2DM [111], DKD [130], and with the risk of T2DM [131]. It has been found that it could have a negative impact on glucose metabolism [119], although some studies have shown opposite results [132,133].

Among the commonly reported findings, an association with T2DM, impaired glucose tolerance, diabetic nephropathy, and inflammatory bowel diseases for the *Fusicatenibacter* [134,135], *Dorea* [123,136,137,138,139,140,141], and the *Ruminococcus torques* groups [140,141,142,143] was found, with some exceptions for *Fusicatenibacter* [111]. In addition, our correlational analysis showed that the *Fusicatenibacter* genus was inversely correlated with FPG levels (in line with a reduction over time of this parameter following NI).

#### 4.2.2. A Significantly Fluctuating Increase in Microbial Taxa in the Short-Term and Mid-Term Follow-Ups

We found a significantly fluctuating increase in some bacterial taxa at several time points (see Supplementary Table S14d online). These taxa included the Tannerellaceae family and its members, *Parabacteroides,* and *P. distasonis* belonging to the Bacteroidota phylum. An almost similar trend was also observed over time for the Burkholderiales order, which showed a significant increase at T12 compared to baseline. Some of the implicated taxa are associated with normoglycemic status [144], metabolic and IR improvements [145,146,147], and normal body weight [148] and are also negatively associated with obesity [27] and T2DM models [149]. In addition, other low-calorie interventions were associated with a significant increase in the Tannerellaceae family, in the *Parabacteroides* genus, and in the *P. distasonis* species in patients with T2DM [150], while the VLCKD was associated with a significant increase in *Parabacteroides* in patients with obesity [151].

#### 4.2.3. A Significant Decrease in Some Microbial Taxa in the Mid-Term (at T6) Compared with Baseline

A positive impact of VLCKD-MD in the mid-term due to the reduction in the *Bilophila* genus and the unclassified *Genus Bilophila* species was found (see Supplementary Table S14e online). These taxa, or species belonging to this genus, are associated with a worsening in clinical parameters, both in patients with T2DM [152] and in mice [153]. In contrast, Sun Y et al. observed the dominance of several bacterial taxa, including the *Bilophila* genus, in controls compared to the T2DM group [146]. Furthermore, a beneficial effect of VLCKD-MD was found for the reduction up to T6 in Negativicutes, which was positively associated with T2DM [136] and diabesity [104].

### 4.3. Beneficial and Detrimental Effect of VLCKD-MD on Microbial Taxa in the Long-Term (After T6)

After the sixth month, most markers of human health were no longer significantly increased compared with baseline, as discussed above. However, both a beneficial and detrimental modulatory effect of VLCKD-MD on the change in a few other bacterial taxa were observed (see Supplementary Table S14g online).

The beneficial modulatory effect was correlated to a reduction in the Lachnospiraceae family, which maintained almost constant levels up to T6 and a decrease at T12 compared with T0. In fact, although Lachnospiraceae are known as beneficial bacteria, they have been associated with metabolic diseases in both human and animal studies as well as with obesity, and, at the same time, an increased production of SCFAs and metabolites produced by members of this family has been associated with gut dysbiosis and obesity [154,155,156,157].

Moreover, after T6, a significant reduction in the Firmicutes phylum and related taxa in the Actinobacteriota phylum, as well as the significant increase in *Parabacteroides distasonis* and the *Burkholderiales* order, was found, as discussed above.

However, the detrimental modulatory effect was related to a reduction in the Coriobacteriia class, the Coriobacteriales order, and the Eggerthellaceae family (Actinobacteriota phylum) at T12 compared with T0 and T6. In fact, it was observed that the Coriobacteriaceae family was associated with good metabolic health in subjects who were overweight and those with obesity, compared to a group of metabolically unhealthy patients with the same disease [158]. From Spearman’s analysis, we consistently observed a negative correlation between Eggerthellaceae and FPG at baseline. Moreover, an increase in Coriobacteriaceae and Eggerthellaceae was associated with polyphenol intake in mice with obesity [159], and the Eggerthellaceae family has been shown to be a key factor related to BW loss and the restoration of intestinal histomorphology [159].

### 4.4. Limited Impact of VLCKD-MD on Microbial Taxa at Specific Time Points, Which Needs Further Investigation

Some changes in microbial taxa, especially in the short-term (see Supplementary Table S14h online), need further investigation given that the literature reports conflicting data on the role they play in T2DM. The Bacteroidota phylum, together with the Bacteroidales order, decreased significantly at T3 compared with baseline. After T3, there was first a slight non-significant increase at T6 that achieved statistical significance at T12 compared with T6 (as well as for the Bacteroidia class), though with values similar to those reported at baseline. It was observed that in patients with obesity, the Bacteroidetes phylum was negatively correlated with body fat and waist circumference [27]. However, the significant decrease in these parameters up to T6 in the KETO cohort did not confirm the increase in the Bacteroidota phylum and related taxa. The reduction in Bacteroidota in the KETO cohort could be due to the decrease in the daily percentage of carbohydrate intake to the limit of significance in KETO at T3 as they encode many carbohydrate-degrading enzymes [160]. The carbohydrate metabolism pathway was significantly increased (for which no significant alterations were found at T2 and T3) at T12 compared to T6.

At a higher taxonomic resolution, several genera and species, including the *Bacteroides* and *Alistipes* genera, followed a nearly identical pattern over time. The abundance of these taxa compared to baseline is restored after T12. The abundance of the *Alistipes shahii* species, the unclassified *Genus Alistipes,* and *Genus Bacteroides* species was reduced up to T6, although significant only at some time points. As for the significance of these variations, growing evidence on the potential contributing or protective role in T2DM or other pathologies, and on the impact in IR, is conflicting [103,111,129,150,152,161,162,163,164,165]. In this study, we showed a significant negative correlation between *A. shahii* and total cholesterol levels, with the Mediterranean diet score and with daily sitting time.

Other taxa belonging to the Firmicutes phylum (the *Subdoligranulum* and *Anaerostipes* genera) and to the Bacteroidota phylum (*Butyricimonas* and *Barnesiella* genera) showed a significant reduction at T2 or T3 in the KETO group [51]. We have not confirmed, in the mid- and long-term follow-ups, a significant impact on these bacterial taxa. However, although statistical significance is not maintained over time, their abundance remains below baseline. It should be noted that the growing literature on *Subdoligranulum* [111,166,167,168,169,170], *Anaerostipes* [111,135,171,172,173,174], *Butyricimonas* [48,129,139,175,176,177], and *Barnesiella* [175,178,179] presents rather conflicting results regarding the implications that these taxa have for T2DM and other diseases. Therefore, further investigation is needed to clarify their physiological role. In this study, we observed that *Subdoligranulum* was significantly and directly related to FPG levels; *Anaerostipes* was inversely correlated with MD; *Butycirimonas* was significantly and negatively correlated with daily sitting time score, and *Barnesiella* was negatively correlated with total cholesterol levels.

Consistent with the taxonomic analysis, only in the KETO group did we find several metabolic pathways that changed significantly over time. In particular, the strongest associations were positively correlated with steroid and carotenoid biosynthesis, with a significant increase over time at T2, T3, and T6 compared to baseline, which was significantly reduced after the sixth month of NI to T12. In addition, the non-homologous end-joining (NHEJ) pathway followed a similar trend.

Carotenoids are molecules with antioxidant and nutraceutical functions that are beneficial for human health [180]. The NHEJ pathway constitutes a type of double-stranded DNA repair (DSB) pathway predominant in human cells that prevents genomic instability, whose deregulation may promote carcinogenesis [181]. Regarding steroid hormones, the excitation of the steroid pathway has been observed to enhance GABA synthesis and GABAergic synaptic transmission by reducing neuronal excitability [182,183]. Consistently, several studies have shown that KD reduces affective disorders and improves levels of social and physical activity [184].

Several pathways were significantly reduced over time in the KETO group; the strongest associations were observed for carbohydrate digestion and absorption, penicillin and cephalosporin biosynthesis, amoebiasis, and dioxin degradation at T6 compared to baseline. Among these, amoebiasis and carbohydrate metabolism were significantly increased at T12 compared to T6. A similar trend was found for Phenylalanine metabolism, which was reduced at T3 compared to T2, at T3 compared to baseline, and at T6 compared to T0, while after the sixth month of NI, it was significantly increased. It should be noted that aromatic amino acids, such as phenylalanine, were found to be higher in the serum of children with obesity [185].

## 5. Conclusions

Current evidence suggests that in patients with diabesity, both the VLCKD and the MD protocols can be beneficial in the medium and long-term. However, the choice between these approaches should be evaluated by expert practitioners [186] with the long-term goal of a personalized treatment that allows patients to achieve the best clinical outcomes [187]. Targeting the GM, which plays a key role in the pathogenesis of obesity and T2DM, through dietary intervention has been proposed as a potential therapeutic approach for diabesity [188].

In this study, the VLCKD-MD has shown greater improvements than the MD on anthropometric and metabolic parameters, as well as a more beneficial impact on the GM phenotype of drug-naïve patients with diabesity.

The improvements have been observed, especially up to the sixth month of NI, both for anthropometric and metabolic parameters and for the taxonomic phenotype.

Based on these results, the VLCKD, when used in conjunction with the MD, could be a valid approach for managing newly diagnosed T2DM without pharmacological intervention. This approach has shown significant improvements in metabolic, anthropometric, and clinical parameters, as well as in physical and mental health status. Additionally, it has a beneficial impact on the gut microbiota phenotype.

## 6. Limitation of the Study

A major limitation of this study is the small sample size, which may have affected the ability to detect significant associations. Since the number of microbiota features to be tested could not be predicted a priori, we did not perform a formal power analysis. The final sample size was determined by participant availability and the resources allocated to the project. Larger studies are needed to validate and further explore our findings.

Similarly, a larger sample size is needed to confirm the findings on the impact of the MD on GM dynamics in the same category of patients. Our future objectives are directed towards defining the key markers associated with T2DM, through the study of the metabolome, and understanding whether these markers can be modulated through dietary interventions, with a view towards personalized therapy.

## Figures and Tables

**Table 1:** Anthropometric, metabolic, lifestyle, and health status in KETO and MEDI groups at T0 and T6.

		KETO			MEDI	
Variable	T0	T6	*p*-Value	T0	T6	*p*-Value
Body weight (kg)	95.5 ± 16.9	79.8 ± 15.5	**0.0048**	86.7 ± 16.8	81.9 ± 16.9	**0.0013**
BMI (kg/m^2^)	34.2 ± 3.6	28.4 ± 1.2	**0.0062**	30.2 ± 4.2	28.5 ± 4.4	**0.0008**
WC (cm)	115.3 ± 7.3	99.4 ± 5.5	**0.0040**	109 ± 8.6	103.8 ± 7.8	**0.0486**
FM (%)	36.8 ± 7.9	28.7 ± 8.8	**0.0120**	34.8 ± 8.5	30.2 ± 9	**0.0060**
FFM (kg)	60.9 ± 16.7	57.9 ± 16.6	**0.0025**	56.5 ± 12.3	56.9 ± 12.6	0.6799
phA (°)	6.2 ± 0.4	6.2 ± 0.7	0.9122	6.2 ± 0.4	6.2 ± 0.7	0.8807
FPG (mg/dL)	135.4 ± 25.1	108.6 ± 10.5	0.1380	133.8 ± 10.2	117.4 ± 9.6	0.0988
HbA1c (%)	6.7 ± 0.9	5.5 ± 0.6	**0.0201**	7.1 ± 0.9	6.2 ± 0.7	0.0675
Total cholesterol (mg/dL)	220 ± 73	181.6 ± 52	0.1738	219.2 ± 11.6	203.8 ± 20.9	0.2517
LDL cholesterol (mg/dL)	141.4 ± 62.8	115.2 ± 46.2	0.2635	139.8 ± 12.8	140.2 ± 28.8	0.9791
HDL cholesterol (mg/dL)	46.8 ± 12.4	47.2 ± 9.7	0.9075	53.4 ± 9.2	57.00 ± 14.44	0.5486
Triglycerides (mg/dL)	158 ± 86.8	95.4 ± 43.1	**0.0484**	130.4 ± 73.1	117.20 ± 66.2	0.1498
SBP (mmHg)	129.2 ± 14.3	135 ± 15	0.3117	151 ± 11.4	145.00 ± 15.00	0.3239
DBP (mmHg)	78.2 ± 10.1	82 ± 6.7	0.4931	81 ± 10.8	81.40 ± 19.74	0.3239
Energy intake (kcal/day)	1738 ± 221	1286 ± 292	0.0669	1840 ± 280	1508 ± 160	0.0511
MDS	24.8 ± 7.8	28.6 ± 2.5	0.3869	26.8 ± 4.6	33 ± 3.16	0.0508
PAL (METs/week)	977 ± 1329.7	1080 ± 622.1	0.7856	708 ± 559.9	1056.00 ± 866.05	0.5611
Daily sitting time (h/day)	6.2 ± 2.5	6.2 ± 3	1.0000	7.2 ± 2.7	6.20 ± 4.21	0.6749
SF-36 PCS	43.2 ± 8.6	54.4 ± 1.3	**0.0477**	50.4 ± 4.2	49.2 ± 6.61	0.5226
SF-36 MCS	47 ± 13.1	53.40 ± 8.1	0.1416	49 ± 15	43.2 ± 10.8	0.2698

Data are presented as mean ± standard deviation (SD). Bold values denote statistical significance at the *p* < 0.05 level. BMI = body mass index; WC = waist circumference; FM = fat mass; FFM; free fat mass; phA (°)= phase angle; FPG = fasting plasma glucose; HbA1c = glycosylated hemoglobin; SBP = systolic blood pressure; DBP = diastolic blood pressure; MDS = Mediterranean diet score; PAL = physical activity level; METs/week = metabolic equivalent of task-minutes per week; SF-36 PCS = physical component summary of SF-36; SF-36 MCS = mental component summary of SF-36. KETO = patients who have sequentially followed a very-low-calorie ketogenic diet and a Mediterranean diet (VLCKD-MD); MEDI = patients who followed a low-calorie Mediterranean diet (MD). Samples were analyzed at baseline and after six months (T6) of nutritional intervention.

**Table 2:** Anthropometric, metabolic, lifestyle, and health status in KETO and MEDI groups at T6 and T12.

		KETO			MEDI	
Variable	T6	T12	*p*-Value	T6	T12	*p*-Value
Body weight (kg)	83.6 ± 13.6	88.1 ± 12.9	0.1168	85.8 ± 16.6	87.7 ± 17.9	0.1544
BMI (kg/m^2^)	28 ± 1	29.6 ± 0.5	0.1170	29.1 ± 4.8	29.7 ± 4.9	0.1108
WC (cm)	99.2 ± 6.3	103.7 ± 5.2	**0.0324**	104.7 ± 8.7	107.7 ± 7.5	0.1727
FM (%)	25.6 ± 6.3	30.2 ± 7.1	**0.0079**	29.3 ± 10.2	31.5 ± 11.7	0.0890
FFM (kg)	62.8 ± 14.5	62.1 ± 13.6	0.5931	60.1 ± 11.8	59.8 ± 12.7	0.7883
phA (°)	6.3 ± 0.7	6.5 ± 0.9	0.5158	5.5 ± 0.3	6.1 ± 0.3	**0.0246**
FPG (mg/dL)	109.5 ± 12	108 ± 15.9	0.7431	115.2 ± 9.6	132.7 ± 17.5	0.0911
HbA1c (%)	5.6 ± 0.6	5.8 ± 0.6	0.1411	6.5 ± 0.1	6.3 ± 0.3	0.5636
Total cholesterol (mg/dL)	171 ± 53.4	189 ± 56.3	**0.0437**	195.5 ± 11.1	231.7 ± 32.7	**0.0444**
LDL cholesterol (mg/dL)	104.2 ± 45.2	123.2 ± 48.3	**0.0295**	116.5 ± 15	137.2 ± 32.3	0.1700
HDL cholesterol (mg/dL)	46.5 ± 11	49.7 ± 9.5	0.4213	53 ± 13.1	66 ± 14.4	**0.0031**
Triglycerides (mg/dL)	101 ± 47.5	80 ± 38.5	0.2705	129.7 ± 69.2	134.3 ± 73.8	0.4351
SBP (mmHg)	128.7 ± 6.29	142.5 ± 8.7	0.1152	143.7 ± 17	142.5 ± 17.5	0.9211
DBP (mmHg)	80 ± 5.8	92.5 ± 2.9	**0.0305**	78.7 ± 21.7	82.5 ± 15.6	0.6376
MDS	28.7 ± 2.8	33.3 ± 2.2	0.0780	33 ± 3.6	30.2 ± 11.1	0.6909
PAL (METs/week)	1192.5 ± 657	1218.7 ± 989.4	0.9346	1240 ± 880	932.5 ± 380.8	0.5488
Daily sitting time (h/day)	6.7 ± 3.1	6.6 ± 1.5	0.6042	5.5 ± 4.5	7 ± 3.6	0.5472
SF-36 PCS	54.7 ±1.3	44 ± 8	0.0820	48.7 ± 7.1	45.3 ± 8	**0.0377**
SF-36 MCS	55.5 ± 7.6	57 ± 5.7	0.4765	45 ± 6.2	46.3 ± 6	0.8605

Data are presented as mean ± standard deviation (SD). Bold values denote statistical significance at the *p* < 0.05 level. BMI = body mass index; WC = waist circumference; FM = fat mass; FFM; free fat mass; phA (°) = phase angle; FPG = fasting plasma glucose; HbA1c = glycosylated hemoglobin; SBP = systolic blood pressure; DBP = diastolic blood pressure; MDS = Mediterranean diet score; PAL = physical activity level; METs/week = metabolic equivalent of ask-minutes per week; SF-36 PCS = physical component summary of SF-36; SF-36 MCS = mental component summary of SF-36. KETO = patients who have sequentially followed a very-low-calorie ketogenic diet and a Mediterranean diet (VLCKD-MD); MEDI = patients who followed a low-calorie Mediterranean diet (MD). Samples were analyzed after six (T6) and twelve (T12) months of nutritional intervention.

**Table 3:** Anthropometric, metabolic, lifestyle, and health status in KETO and MEDI groups at T0 and T12.

		KETO			MEDI	
Variable	T0	T12	*p*-Value	T0	T12	*p*-Value
Body weight (kg)	98.8 ± 17.6	88.1 ± 12.9	0.1535	90.9 ± 16	87.7 ± 17.9	0.0534
BMI (kg/m^2^)	33.1 ± 3.2	29.6 ± 0.5	0.1273	30.9 ± 4.4	29.7 ± 4.9	**0.0445**
WC (cm)	114.4 ± 8.1	103.7 ± 5.2	0.0845	111.5 ± 7.6	107.7 ± 7.5	0.2251
FM (%)	34.1 ± 5.9	30 ± 7.3	0.2601	34.6 ± 9.9	31.5 ± 11.7	0.1223
FFM (kg)	65.6 ± 15.1	62.1 ± 13.6	0.0571	59.1 ± 12.4	59.8 ± 12.7	**0.0416**
phA (°)	6.2 ± 0.5	6.5 ± 0.9	0.4970	5.5 ± 0.5	6.1 ± 0.3	0.0681
FPG (mg/dL)	140.2 ± 26.1	108 ± 15.9	0.1695	137 ± 8.4	132.7 ± 17.5	0.5995
HbA1c (%)	6.7 ± 1.1	5.8 ± 0.6	0.1177	7.3 ± 0.9	6.4 ± 0.3	0.2320
Total cholesterol (mg/dL)	219.5 ± 84.4	189 ± 56.3	0.3120	221.7 ± 11.6	231.7 ± 32.7	0.5258
LDL cholesterol (mg/dL)	136.5 ± 71.4	123.2 ± 48.3	0.6516	143.2 ± 11.7	137.2 ± 32.4	0.7044
HDL cholesterol (mg/dL)	47.7 ± 14.1	49.7 ± 9.5	0.5561	49.7 ± 4.8	66 ± 14.4	0.1119
Triglycerides (mg/dL)	175 ± 90.1	80 ± 38.5	0.0939	144.3 ± 76.4	134.3 ± 73.8	0.1311
SBP (mmHg)	124 ± 9.7	142.5 ± 8.7	0.0593	151.3 ± 13.2	142.5 ± 17.6	0.2560
DBP (mmHg)	76.5 ±10.8	92.5 ± 2.9	**0.0377**	78.8 ± 11.1	82.5 ± 15.6	0.7487
Energy intake (kcal/day)	1738 ± 221	1125 ± 75	**0.0493**	1840 ± 280	1322 ± 302	0.1183
MDS	26.5 ± 7.9	33.3 ± 2.2	0.1253	28 ± 4.3	30.3 ± 11.1	0.7076
PAL (METs/week)	1022.5 + 1531	1219.3 ± 990.2	0.6543	465 ± 155.9	932.5 ± 380.8	0.1763
Daily sitting time (h/day)	6.8 ± 2.5	6.3 ± 1.5	0.6638	8 ± 2.3	7 ± 3.6	0.2522
SF-36 PCS	43 ± 9.9	44 ± 8	0.8311	49 ± 2.7	45.3 ± 8	0.3802
SF-36 MCS	50.5 ± 12.2	57 ± 5.7	0.1603	55 ± 5.3	46.3 ± 6	**0.0102**

Data are presented as mean ± standard deviation (SD). Bold values denote statistical significance at the *p* < 0.05 level. BMI = body mass index; WC = waist circumference; FM = fat mass; FFM; free fat mass; phA (°)= phase angle; FPG = fasting plasma glucose; HbA1c = glycosylated hemoglobin; SBP = systolic blood pressure; DBP = diastolic blood pressure; MDS = Mediterranean diet score; PAL = physical activity level; METs/week = metabolic equivalent of task-minutes per week; SF-36 PCS = physical component summary of SF-36; SF-36 MCS = mental component summary of SF-36. KETO = patients who have sequentially followed a very-low-calorie ketogenic diet and a Mediterranean diet (VLCKD-MD); MEDI = patients who followed a low-calorie Mediterranean diet (MD). Samples were analyzed at baseline and after twelve months (T12) of nutritional intervention.

## Data Availability

The original data presented in the study are openly available in the European Nucleotide Archive (ENA) (https://www.ebi.ac.uk/ena) and under the accession number PRJEB82367 (ERA30932128) at https://www.ebi.ac.uk/ena/browser/view/PRJEB82367 (accessed on 10 November 2024).

## Supplementary Materials

The following supporting information can be downloaded at: https://www.mdpi.com/article/10.3390/metabo15010022/s1. Table S1: Anthropometric, metabolic, lifestyle, and health status in KETO and MEDI groups at T3 and T6; Table S2: Anthropometric, metabolic, lifestyle, and health status T3–T6 variations in KETO and MEDI groups; Table S3: Anthropometric, metabolic, lifestyle, and health status T0–T6 variations in KETO and MEDI groups; Table S4: Anthropometric, metabolic, lifestyle, and health status T6–T12 variations in KETO and MEDI groups; Table S5: Anthropometric, metabolic, lifestyle and health status in KETO and MEDI groups at T0 and T12; Table S6: Alpha diversity analysis between KETO and MEDI; Table S7: Alpha diversity analysis in KETO over time; Table S8: Alpha diversity analysis in MEDI over time; Table S9: GM beta diversity analysis between KETO and MEDI; Table S10: GM beta diversity analysis in KETO over time; Table S11: GM beta diversity analysis in MEDI over time; Table S12: Firmicutes/Bacteroidota ratio analysis in KETO over time; Table S13: Firmicutes/Bacteroidota ratio analysis in MEDI over time; Table S14: Changes in relative abundance of gut microbiota taxa over time in the KETO group; Table S15: Spearman correlation analysis between GM alterations and clinical and nutritional variables in patients with diabesity at baseline. Figure S1: Principal coordinate analysis plots showing gut microbiota compositional changes along time points for each diet; Figure S2: Gut microbiota Firmicutes/Bacteoroidota ratio comparison between time points for each diet; Figure S3: Significant changes in gut microbiota taxa abundance between time points in KETO and MEDI cohorts; Figure S4: Graphical changes in gut microbiota taxa abundance over time in KETO and MEDI groups.
