# Supplementary material for: Impact of Ketogenic and Mediterranean Diets on Gut Microbiota Profile and Clinical Outcomes in Drug-Naïve Patients with Diabesity: A 12-Month Pilot Study
Source: Metabolites. 2025 Jan 6;15(1):22. doi: 10.3390/metabo15010022 (PMC11766981; doi:10.3390/metabo15010022)
Supplement: Supplementary file 1 [file metabolites-15-00022-s001.zip › metabolites-3337647-supplementary.pdf]

Supplementary Materials

Impact of Ketogenic and Mediterranean Diets on Gut Microbiota Profile and Clinical Outcomes in Drug-Naïve Patients with Diabesity: a 12-Months Pilot Study

Vanessa Palmas [1†], Andrea Deledda [2†], Vitor Heidrich [3,4], Giuseppina Sanna [1], Giulia Cambarau [2], Michele Fosci [5], Lorenzo Puglia [5], Enrico Antonio Cappai [2], Alessio Lai [6], Andrea Loviselli [5], Aldo Manzin [1*] and Fernanda Velluzzi [2]

[1] Department of Biomedical Sciences, University of Cagliari, 09042, Monserrato, Italy; V.P.: vanessa.pamas@unica.it; G.S.: g.sanna@unica.it; A.M.: aldo.manzin@unica.it

[2] Department of Medical Sciences and Public Health, Obesity Unit, University of Cagliari, 09124 Cagliari, Italy; A.D.: andredele@tiscali.it; G.C.: giuliacambarau@tiscali.it; E.A.C.: cappai42@gmail.com; F.V.: fernanda.velluzzi@unica.it

[3] Departamento de Bioquímica, Instituto de Química, Universidade de São Paulo, 05508-900, São Paulo, Brazil; vheidrich@mochsl.org.br

[4] Centro de Oncologia Molecular, Hospital Sírio-Libanês, 01308-050, São Paulo, Brazil; vheidrich@mochsl.org.br

[5] Department of Medical Sciences and Public Health, Endocrinology Unit, University of Cagliari, 09042 Monserrato, Italy; M.F.: michele.fosci@aslsulcis.it; L.P.: lorenzo.puglia02@icatt.it; A.L.: alovise2@gmail.com

[6] Diabetologia, P.O. Binaghi, ASSL Cagliari, 09126 Cagliari, Italy; alexlai1@tiscali.it

* Correspondence: aldo.manzin@unica.it

† These authors contributed equally to the work.

Table S1. Anthropometric, metabolic, lifestyle and health status in KETO and MEDI groups at T3 and T6.

	KETO			MEDI		
**Variable**	**T3**	**T6**	***p*-value**	**T3**	**T6**	***p*-value**
Body weight (kg)	81.2±13.7	79.8±15.5	0.4916	83.6±18.3	81.9±16.9	0.0520
BMI (kg/m^2)	29±2.6	28.4±1.2	0.3920	29.1±4.8	28.5±4.4	**0.0354**
WC (cm)	102.2±7.9	99.4±5.5	0.3923	104.3±9.5	103.8±7.8	0.8038
FM (%)	29.6±9	28.7±8.8	0.3653	31.8±9.6	30.2±9	**0.0295**
FFM (kg)	58.0±16	57.9±16.6	0.8563	56.7±13.2	56.9±12.6	0.7123
phA (°)	5.9±0.5	6.2±0.7	0.1979	5.9±0.6	5.6±0.3	0.3432
FPG (mg/dl)	107.2±15.5	108.6±10.5	0.8718	140.6±23.5	117.4±9.6	0.1385
HbA1c (%)	5.4±0.5	5.5±0.6	0.2355	6.4±0.8	6.2±0.7	0.3868
Total Cholesterol (mg/dl)	159.4±38.9	181.6±52	**0.0331**	210.2±20.7	203.8±20.9	0.6038
LDL Cholesterol (mg/dl)	101±32.3	115.2±46.2	0.0988	124.2±12.4	123.2±19.8	0.9112
HDL Cholesterol (mg/dl)	46.8±8.5	47.2±9.6	0.7174	59.6±8.4	57±14.4	0.4704
Triglycerides (mg/dl)	58.8±19.2	95.4±43	0.2020	131.6±96.4	117.2±66.2	0.3866
SBP (mmHg)	137 ±16.8	135±15	0.6483	147±17.2	145±15	0.7943
DBP (mmHg)	83±7.6	82±6.7	0.7990	85±13.3	81.4±19.7	0.3046
Energy Intake (kcal/day)	1143±224	1286±292	0.2959	1630±104	1508±160	0.2383
MDS	27.8±2.1	28.1±2.5	0.4954	30.4±5.6	33.0±3.2	0.5317
PAL (METs/week)	1018±551.3	1080±622.1	0.8570	754±501	1056±866.1	0.5413
Daily sitting time (h/day)	5.8±2.1	6.2±3	0.5870	6.6±3.3	6.2±4.2	0.7780
SF-36 PCS	49.6±6.7	54.4±1.3	0.1875	46.6±8.1	49.2±6.6	0.5162
SF-36 MCS	55±9.2	53.4±8.1	0.5795	40.8±9.6	43.2±10.8	0.5722

Data are presented as mean ± standard deviation (SD). Bold values denote statistical significance at the $p < 0.05$ level. BMI = Body Mass Index; WC = Waist Circumference; FM = Fat Mass; FFM = Free Fat Mass; phA (°) = phase Angle; FPG = fasting plasma glucose; HbA1c = glycosylated hemoglobin; SBP = Systolic Blood Pressure; DBP = Diastolic Blood Pressure; MDS = Mediterranean Diet Score; PAL = Physical Activity Level; METs/week = Metabolic Equivalent of Task-minutes per week; SF-36 PCS = Physical Component Summary of SF-36; SF-36 MCS = Mental Component Summary of SF-36. KETO = patients who followed a very-low-calorie ketogenic diet (VLCKD); MEDI = patients who followed a low-calorie Mediterranean diet (MD). Samples were analyzed after three (T3) and six months (T6) of nutritional intervention.

Table S2. Anthropometric, metabolic, lifestyle, and health status T3–T6 variations in KETO and MEDI groups

Variable (delta T3-T6)			
**Variable**	**KETO**	**MEDI**	***p*-value**
Body weight (kg)	-1.4±4.1	-1.7±1.4	0.8513
BMI (kg/m²)	-0.6±1.4	-0.4±0.5	0.8290
WC (cm)	-2.8±6.5	-0.5±4.2	0.5269
FM (%)	-0.8±2.1	-1.6±1.1	0.4722
FFM (kg)	-0.2±1.8	0.2±1.12	0.7203
phA (°)	0.4±0.5	0.2±0.7	0,2229
FPG (mg/dl)	1.6 ±10.9	-23.2±28.1	0.1029
HbA1c (%)	0.12±0.19	-0.2±0.5	0.1985
Total Cholesterol (mg/dl)	22.2±15.5	-6.4±25,4	0.0643
LDL Cholesterol (mg/dl)	14.2±14.8	-1±18.8	0.1938
HDL Cholesterol (mg/dl)	0.4±2.3	-2.6±7.3	0.4064
Triglycerides (mg/dl)	36±53.9	-14.4±33.2	0.1127
SBP (mmHg)	-2±9.1	-2±16	1.000
DBP (mmHg)	-1±8.2	- 3.6 ±6.8	0.6014
MDS	0.8±2.4	2.6±8.5	0.6607
PAL (METs/week)	62±721.5	302 ±1012.6	0.6774
Daily sitting time (h/day)	0.4±1.5	-0.4±3	0.6059
SF-36 PCS	4.8±6.8	2.6±8.2	0.6551
SF-36 MCS	-1.6±5.9	2.4±8.7	0.4218

Data are presented as mean ± standard deviation (SD). *p* equal to or less than 0.05 was considered statistically significant. BMI = Body Mass Index; WC = Waist Circumference; FM = Fat Mass; FFM; Free Fat Mass; phA (°)= phase Angle; FPG = fasting plasma glucose; HbA1c = glycosylated hemoglobin; SBP = Systolic Blood Pressure; DBP = Diastolic Blood Pressure; MDS = Mediterranean Diet Score; PAL = Physical Activity Level; METs/week = Metabolic Equivalent of Task-minutes per week; SF-36 PCS = Physical Component Summary of SF-36; SF-36 MCS = Mental Component Summary of SF-36. KETO= patients who followed a very-low-calorie ketogenic diet (VLCKD); MEDI= patients who followed a low-calorie Mediterranean diet (MD). Samples were analyzed after three (T3) and after six (T6) months of nutritional intervention.

Table S3. Anthropometric, metabolic, lifestyle, and health status T0–T6 variations in KETO and MEDI groups

	Variable (delta T0-T6)		
**Variable**	**KETO**	**MEDI**	***p*-value**
Body weight (kg)	-15.7±6.2	-4.8±1.3	**0.0049**
BMI (kg/m^2)	-5.8±2.4	-1.7±0.4	**0.0065**
WC (cm)	-15.9±6	-5.2±4.1	**0.0110**
FM (%)	-8.2±4.2	-4.6±1.9	0.1244
FFM (kg)	-3.1±1	0.4±2	**0.0089**
phA (°)	0.04±0.6	0.02±0.5	0.9550
FPG (mg/dl)	-26.8±32.4	-16.4±17.2	0.5436
HbA1c (%)	-1.2±0.7	-0.9±0.8	0.6141
Total Cholesterol (mg/dl)	-38.4±52	-15.4±25.7	0.4010
LDL Cholesterol (mg/dl)	-26.2±45	0.4±29	0.2987
HDL Cholesterol (mg/dl)	0.4±7.2	3.6±12.3	0.6295
Triglycerides (mg/dl)	-62.6±50	-11.2±16.8	0.0604
SBP (mmHg)	5.8±11.2	-6±12	0.1458
DBP (mmHg)	3.8±11.3	0.4±18	0.7302
MDS	3.8±8.8	6.2±5	0.6094
PAL (METs/week)	103±791.7	348±1229.3	0.7176
Daily sitting time (h/day)	0±2.2	-1±5	0.6914
SF-36 PCS	11.2±8.9	-1.2±2.8	**0.0209**
SF-36 MCS	6.4±7.8	-5.8±10.1	0.0658

Data are presented as mean ± standard deviation (SD). Bold values denote statistical significance at the $p < 0.05$ level. BMI = Body Mass Index; WC = Waist Circumference; FM = Fat Mass; FFM; Free Fat Mass; phA (°)= phase Angle; FPG = fasting plasma glucose; HbA1c = glycosylated hemoglobin; SBP = Systolic Blood Pressure; DBP = Diastolic Blood Pressure; MDS = Mediterranean Diet Score; PAL = Physical Activity Level; METs/week = Metabolic Equivalent of Task-minutes per week; SF-36 PCS = Physical Component Summary of SF-36; SF-36 MCS = Mental Component Summary of SF-36. KETO= patients who followed a very-low-calorie ketogenic diet (VLCKD); MEDI= patients who followed a low-calorie Mediterranean diet (MD). Samples were analyzed at baseline and after six months (T6) of nutritional intervention.

Table S4. Anthropometric, metabolic, lifestyle, and health status T6–T12 variations in KETO and MEDI groups

Variable (delta T6-T12)			
Variable	KETO	MEDI	*p*-value
Body weight (kg)	4.3±3.9	1.9±2	0,3138
BMI (kg/m²)	1.5±1.4	0.6±0,5	0.2572
WC (cm)	4.5±2.4	3 ±3.4	0.4943
FM (%)	4.6±1.4	2.6 ± 1.7	0.0746
FFM (kg)	-0.7±2.3	-0.8±2.2	0.9595
phA (°)	0.1±0.3	0.6±0.4	0.1094
FPG (mg/dl)	-1.5±8.3	17.5±14.2	0.0610
HbA1c (%)	0.1±0.1	-0.1±0.4	0.2644
Total Cholesterol (mg/dl)	18±10.7	36.2±21.2	0.1824
LDL Cholesterol (mg/dl)	19±10.8	20.7±23	0.8389
HDL Cholesterol (mg/dl)	3.2±7	13±2.9	**0.0424**
Triglycerides (mg/dl)	-21 ±31.2	4.5±10	0.1703
SBP (mmHg)	13.7±12.5	-1.2±23.2	0.2988
DBP (mmHg)	12.5±6.4	3.7±14.4	0.3089
MDS	4.5±3.4	-2.7±12.5	0.3077
PAL (METs/week)	26.3 ±589.5	-232.5±793.3	0.6193
Daily sitting time (h/day)	-0.5±1.7	1.5±4.4	0.4330
SF-36 PCS	-10.8±8.3	-3.3 ± 1.2	0.1956
SF-36 MCS	1.5±3.7	1.33 ± 11.6	0.9790

Data are presented as mean ± standard deviation (SD). Bold values denote statistical significance at the $p < 0.05$ level. BMI = Body Mass Index; WC = Waist Circumference; FM = Fat Mass; FFM; Free Fat Mass; phA (°)= phase Angle; FPG = fasting plasma glucose; HbA1c = glycosylated hemoglobin; SBP = Systolic Blood Pressure; DBP = Diastolic Blood Pressure; MDS = Mediterranean Diet Score; PAL = Physical Activity Level; METs/week = Metabolic Equivalent of Task-minutes per week; SF-36 PCS = Physical Component Summary of SF-36; SF-36 MCS = Mental Component Summary of SF-36. KETO= patients who followed a very-low-calorie ketogenic diet (VLCKD); MEDI= patients who followed a low-calorie Mediterranean diet (MD). Samples were analyzed after six (T6) and twelve (T12) months of nutritional intervention.

Table S5. Anthropometric, metabolic, lifestyle and health status in KETO and MEDI groups at T0 and T12

	Variable (delta T0-T12)		
**Variable**	**KETO**	**MEDI**	***p*-value**
Body weight (kg)	-10.8±11.5	-3.1±2	0.2368
BMI (kg/m²)	-3.6±3.5	-1.2±0.7	0.2337
WC (cm)	-10.6±8.4	-3.7±4.9	0.2062
FM (%)	-4.4±5.2	3.1±2.9	0.6896
FFM (kg)	-3.3±2.4	0.7±0.4	**0.0173**
phA (°)	0.3±0.7	0.6±0.4	0.4937
FPG (mg/dl)	-32.2±35.8	-4.2±14.5	0.1975
HbA1c (%)	-0.97±0.9	-0.95±1.3	0.9754
Total Cholesterol (mg/dl)	-30.5±50.3	10±28	0.2088
LDL Cholesterol (mg/dl)	-13.2±53	-6±29	0.8183
HDL Cholestrol (mg/dl)	2±6	16.2±14.6	0.1209
Triglycerides (mg/dl)	-95±78.4	-10± 9.7	0.0749
SBP (mmHg)	18.5±12.5	-8.8±12.5	**0.0215**
DBP (mmHg)	16±9	3.7±21.4	0.3311
MDS	6.7±6.4	2.2±10.9	0.5033
PAL (METs/week)	196.2±795.1	467.5±530.7	0.5910
Daily setting time (h/day)	-0.5±2	-1±1.4	0.7049
SF-36 PCS	1± 8.6	- 3.7 ±5.7	0.4564
SF-36 MCS	6.5±7	-8.7 ± 1.5	**0.0155**

Data are presented as mean ± standard deviation (SD). Bold values denote statistical significance at the $p < 0.05$ level. BMI = Body Mass Index; WC = Waist Circumference; FM = Fat Mass; FFM; Free Fat Mass; phA (°)= phase Angle; FPG = fasting plasma glucose; HbA1c = glycosylated hemoglobin; SBP = Systolic Blood Pressure; DBP = Diastolic Blood Pressure; MDS = Mediterranean Diet Score; PAL = Physical Activity Level; METs/week = Metabolic Equivalent of Task-minutes per week; SF-36 PCS = Physical Component Summary of SF-36; SF-36 MCS = Mental Component Summary of SF-36. KETO= patients who followed a very-low-calorie ketogenic diet (VLCKD); MEDI= patients who followed a low-calorie Mediterranean diet (MD). Samples were analyzed at baseline and after twelve months (T12) of nutritional intervention.

Table S6. Alpha diversity analysis between KETO and MEDI

**Shannon**	***p***
**T0 (KETO *vs.* MEDI)**	0.052
**T2 (KETO *vs.* MEDI)**	0.662
**T3 (KETO *vs.* MEDI)**	0.792
**T6 (KETO *vs.* MEDI)**	0.463
**T12 (KETO *vs.* MEDI)**	0.886
**Observed ASVs**	***p***
**T0 (KETO *vs.* MEDI)**	0.931
**T2 (KETO *vs.* MEDI)**	0.410
**T3 (KETO *vs.* MEDI)**	0.537
**T6 (KETO *vs.* MEDI)**	0.056
**T12 (KETO *vs.* MEDI)**	0.686
**Evenness (Pielou's J)**	***p***
**T0 (KETO *vs.* MEDI)**	**0.017**
**T2 (KETO *vs.* MEDI)**	0.792
**T3 (KETO *vs.* MEDI)**	0.792
**T6 (KETO *vs.* MEDI)**	**0.032**
**T12 (KETO *vs.* MEDI)**	0.343

Differences in the Shannon index (H’), Observed ASVs and evenness (Pielou’s index: J’) between KETO and MEDI were assessed by Mann-Whitney U test using the stats R package. p equal to or less than 0.05 was considered statistically significant. KETO= patients who followed a very-low-calorie ketogenic diet (VLCKD); MEDI= patients who followed a low-calorie Mediterranean diet (MD); pale values= results up to T3 follow-up already published [1]; bright values= new results relating to analyzes extended up to T12.

Table S7. Alpha diversity analysis in KETO over time

Shannon	Median (IQR)	*p* (*vs* T2)	*p* (*vs* T3)	*p* (*vs* T6)	*p* (*vs* T12)
T0	3.77 (0.17)	0.688	0.563	0.813	0.625
T2	3.57 (0.63)		0.438		
T3	3.30 (0.71)			0.892	
**T6**	2.76 (0.61)				1
**T12**	3.77 (0.17)				

Observed ASVs	Median (IQR)	*p* (*vs* T2)	*p* (*vs* T3)	*p* (*vs* T6)	*p* (*vs* T12)
T0	171.5 (23.75)	0.293	0.688	0.125	0.125
T2	200.5 (22.25)		0.219		
T3	190 (22.25)			0.438	
**T6**	186 (10)				1
**T12**	175 (54.75)				

Evenness (Pielou's J)	Median (IQR)	*p* (*vs* T2)	*p* (*vs* T3)	*p* (*vs* T6)	*p* (*vs* T12)
T0	0.74 (0.06)	0.438	0.438	0.125	0.125
T2	0.68 (0.12)		0.563		
T3	0.64 (0.15)			0.438	
**T6**	0.55 (0.10)				0.625
**T12**	0.65 (0.04)				

Differences in the Shannon index (H'), Observed ASVs and evenness (Pielou's index: J') in KETO before (T0), after two (T2), three (T3), six (T6) and twelve (T12) months of very low calorie ketogenic diet were assessed by the paired Wilcoxon signed-rank test using the *stats* R package. *p* equal to or less than 0.05 was considered statistically significant. KETO= patients who followed a very-low-calorie ketogenic diet (VLCKD); pale values= results up to T3 follow-up already published [1]; bright values= new results relating to analyzes extended up to T12. IQR, interquartile range.

Table S8. Alpha diversity analysis in MEDI over time

**Shannon**	**Median (IQR)**	***p* (*vs* T2)**	***p* (*vs* T3)**	***p* (*vs* T6)**	***p* (*vs* T12)**
T0	3.08 (0.42)	0.313	1	0.063	0.250
T2	3.16 (0.70)		0.625		
T3	3.38 (1.09)			0.313	
**T6**	3.93 (0.50)				0.875
**T12**	3.71 (0.97)				
**Observed ASVs**	**Median (IQR)**	***p* (*vs* T2)**	***p* (*vs* T3)**	***p* (*vs* T6)**	***p* (*vs* T12)**
T0	172 (35)	0.625	1	0.438	0.375
T2	194 (24)		1		
T3	180 (9)			0.438	
**T6**	200 (64)				0.625
**T12**	178 (72.75)				
**Evenness (Pielou's J)**	**Median (IQR)**	***p* (*vs* T2)**	***p* (*vs* T3)**	***p* (*vs* T6)**	***p* (*vs* T12)**
T0	0.61 (0.06)	0.188	1	0.063	0.125
T2	0.61 (0.10)		0.625		
T3	0.64 (0.20)			0.313	
**T6**	0.75 (0.06)				1
**T12**	0.70 (0.12)				

Differences in the Shannon index (H’), Observed ASVs and evenness (Pielou’s index: J’) in MEDI before (T0), after two (T2), three (T3), six (T6) and twelve (T12) months of low-calorie Mediterranean diet were assessed by the paired Wilcoxon signed-rank test using the *stats* R package. *p* equal to or less than 0.05 was considered statistically significant. MEDI= patients who followed a low-calorie Mediterranean diet (MD); pale values= results up to T3 follow-up already published [1]; bright values= new results relating to analyzes extended up to T12. IQR, interquartile range.

Table S9. GM beta diversity analysis between KETO and MEDI

Bray-Curtis	T0	T2	T3	T6	T12
**Degrees of freedom**	1	1	1	1	1
**Sum of squares**	0.599	0.308	0.316	0.402	0.298
**Mean of squares**	0.599	0.308	0.316	0.402	0.298
**F**	1.727	0.830	0.848	1.037	0.762
**R^2**	0.161	0.084	0.086	0.115	0.113
p	0.013	0.696	0.656	0.368	0.964
**Unweighted UniFrac**	**T0**	**T2**	**T3**	**T6**	**T12**
**Degrees of freedom**	1	1	1	1	1
**Sum of squares**	0.118	0.125	0.144	0.160	0.140
**Mean of squares**	0.118	0.125	0.144	0.160	0.140
**F**	0.838	0.869	1.079	1.059	0.876
**R^2**	0.085	0.088	0.107	0.117	0.127
p	0.864	0.753	0.357	0.377	0.657
**Weighted UniFrac**	**T0**	**T2**	**T3**	**T6**	**T12**
**Degrees of freedom**	1	1	1	1	1
**Sum of squares**	0.163	0.063	0.047	0.231	0.060
**Mean of squares**	0.163	0.063	0.047	0.231	0.060
**F**	1.724	0.480	0.490	2.680	0.482
**R^2**	0.161	0.051	0.052	0.250	0.075
p	0.150	0.674	0.695	**0.046**	0.789

Differences in the beta diversity index between KETO and MEDI were evaluated by Permutational Multivariate Analysis of Variance (PERMANOVA), performed using the *vegan* R package. Samples were analyzed before (T0) and after two (T2), three (T3), six (T6) and twelve (T12) months of the of nutritional intervention. *p* equal to or less than 0.05 was considered statistically significant. KETO= patients who followed a very low calorie ketogenic diet, MEDI= patients who followed a low-calorie Mediterranean diet; pale values= results up to T3 follow-up already published [1]; bright values= new results relating to analyzes extended up to T12.

Table S10. GM beta diversity analysis in KETO over time

**Bray-Curtis**	**T0 *vs* T2**	**T0 *vs* T3**	**T2 *vs* T3**	**T0 *vs* T6**	**T0 *vs* T12**	**T3 *vs* T6**
**Degrees of freedom**	1	1	1	1	1	1
**Sum of squares**	0.318	0.319	0.080	0.376	0.226	0.109
**Mean of squares**	0.318	0.319	0.080	0.376	0.226	0.109
**F**	0.830	0.832	0.206	0.973	0.577	0.278
**R^2**	0.077	0.077	0.020	0.098	0.067	0.030
p	0.737	0.711	0.958	0.533	0.959	0.960
**Unweighted UniFrac**	**T0 *vs* T2**	**T0 *vs* T3**	**T2 *vs* T3**	**T0 *vs* T6**	**T0 *vs* T12**	**T3 *vs* T6**
**Degrees of freedom**	1	1	1	1	1	1
**Sum of squares**	0.116	0.104	0.057	0.121	0.131	0.052
**Mean of squares**	0.116	0.104	0.057	0.121	0.131	0.052
**F**	0.802	0.758	0.423	0.840	0.860	0.389
**R^2**	0.074	0.070	0.041	0.085	0.097	0.041
p	0.733	0.828	0.934	0.688	0.651	0.990
**Weighted UniFrac**	**T0 *vs* T2**	**T0 *vs* T3**	**T2 *vs* T3**	**T0 *vs* T6**	**T0 *vs* T12**	**T3 *vs* T6**
**Degrees of freedom**	1	1	1	1	1	1
**Sum of squares**	0.166	0.196	0.019	0.316	0.090	0.034
**Mean of squares**	0.166	0.196	0.019	0.316	0.090	0.034
**F**	1.960	2.657	0.208	4.848	1.027	0.460
**R^2**	0.164	0.210	0.020	0.350	0.114	0.049
p	0.121	0.067	0.844	**0.021**	0.397	0.754

Differences in the beta diversity index in KETO before (T0) and after two (T2), three (T3), six (T6) and twelve (T12) months of very low calorie ketogenic diet were evaluated by Permutational Multivariate Analysis of Variance (PERMANOVA), performed using the *vegan* R package. *p* equal to or less than 0.05 was considered statistically significant. KETO= patients who followed a very low calorie ketogenic diet; pale values= results up to T3 follow-up already published [1]; bright values= new results relating to analyzes extended up to T12.

Table S11. GM beta diversity analysis in MEDI over time

**Bray-Curtis**	**T0 *vs* T2**	**T0 *vs* T3**	**T2 *vs* T3**	**T0 *vs* T6**	**T0 *vs* T12**	**T3 *vs* T6**
**Degrees of freedom**	1	1	1	1	1	1
**Sum of squares**	0.099	0.117	0.107	0.241	0.281	0.181
**Mean of squares**	0.099	0.117	0.107	0.241	0.281	0.181
**F**	0.299	0.356	0.305	0.701	0.842	0.494
**R^2**	0.036	0.043	0.037	0.081	0.108	0.058
p	0.927	0.972	0.950	0.759	0.573	0.926
**Unweighted UniFrac**	**T0 *vs* T2**	**T0 *vs* T3**	**T2 *vs* T3**	**T0 *vs* T6**	**T0 *vs* T12**	**T3 *vs* T6**
**Degrees of freedom**	1	1	1	1	1	1
**Sum of squares**	0.048	0.058	0.040	0.095	0.080	0.062
**Mean of squares**	0.048	0.058	0.040	0.095	0.080	0.062
**F**	0.344	0.423	0.284	0.650	0.559	0.417
**R^2**	0.041	0.050	0.035	0.075	0.074	0.049
p	0.949	0.948	1	0.876	0.919	0.904
**Weighted UniFrac**	**T0 *vs* T2**	**T0 *vs* T3**	**T2 *vs* T3**	**T0 *vs* T6**	**T0 *vs* T12**	**T3 *vs* T6**
**Degrees of freedom**	1	1	1	1	1	1
**Sum of squares**	0.039	0.029	0.087	0.086	0.122	0.107
**Mean of squares**	0.039	0.029	0.087	0.086	0.122	0.107
**F**	0.260	0.235	0.622	0.724	0.960	0.972
**R^2**	0.031	0.028	0.072	0.083	0.121	0.108
p	0.768	0.939	0.657	0.609	0.446	0.477

Differences in the beta diversity index in MEDI before (T0) and after two (T2), three (T3), six (T6) and twelve (T12) months of low-calorie Mediterranean diet were evaluated by Permutational Multivariate Analysis of Variance (PERMANOVA), performed using the *vegan* R package. *p* equal to or less than 0.05 was considered statistically significant. MEDI= patients who followed a low-calorie Mediterranean diet; pale values= results up to T3 follow-up already published [1]; bright values= new results relating to analyzes extended up to T12.

Table S12. Firmicutes/Bacteroidota ratio analysis in KETO over time

Firmicutes/Bacteroidota	Median (IQR)	*p* (*vs* T2)	*p* (*vs* T3)	*p* (*vs* T6)	*p* (*vs* T12)
**T0**	1.56 (1.60)	0.844	1	0.813	0.375
**T2**	1.42 (1.19)		0.563		
**T3**	1.97 (0.77)			0.125	
**T6**	1.02 (0.46)				0.125
**T12**	0.83 (0.42)				

Differences in the Firmicutes/Bacteroidota ratio in KETO before (T0) and after two (T2), three (T3), six (T6) and twelve (T12) months of very low calorie ketogenic diet evaluated by the paired Wilcoxon signed-rank test using the *stats* R package. *p* equal to or less than 0.05 was considered statistically significant. KETO= patients who followed a very low calorie ketogenic diet; pale values= results up to T3 follow-up already published [1]; bright values= new results relating to analyzes extended up to T12; IQR, interquartile range.

Table S13. Firmicutes/Bacteroidota ratio analysis in MEDI over time

Firmicutes/Bacteroidota	Median (IQR)	*p* (*vs* T2)	*p* (*vs* T3)	*p* (*vs* T6)	*p* (*vs* T12)
**T0**	1.56 (1.27)	0.625	0.625	0.813	0.625
**T2**	1.06 (0.90)		0.625		
**T3**	2.97 (0.58)			0.625	
**T6**	1.60 (0.83)				0.625
**T12**	1.24 (1.18)				

Differences in the Firmicutes/Bacteroidota ratio in MEDI before (T0) and after two (T2), three (T3), six (T6) and twelve (T12) months of very low calorie Mediterranean diet evaluated by the paired Wilcoxon signed-rank test using the *stats* R package. *p* equal to or less than 0.05 was considered statistically significant. MEDI= patients who followed a very low calorie Mediterranean diet. pale values= results up to T3 follow-up already published [1] bright values= new results relating to analyzes extended up to T12; IQR, interquartile range.

Table S14. Changes in relative abundance of gut microbiota taxa over time in the KETO group

a) Significant Increase Over Time in Several Microbial Taxa from Baseline up to T6 and reduction at T12 Compared with T6

Phylum	Class	Order	Family	Genus	Species	Ref. Group	*p*	*q*	↓/↑	Coeff.	Std.Err.
Verrucomicrobiota						T0–T2	0.014	0.120	↑	5.07	1.70
Verrucomicrobiota						T0–T3	0.020	0.120	↑	4.69	1.70
Verrucomicrobiota						T0_T6	0.004	0.047	↑	6.34	1.90
Verrucomicrobiota						T6–T12	0.016	0.077	↓	–5.63	2.10
	Verrucomicrobiae					T0–T2	0.014	0.162	↑	5.06	1.70
	Verrucomicrobiae					T0–T3	0.020	0.162	↑	4.69	1.70
	Verrucomicrobiae					T0–T6	0.004	0.042	↑	6.33	1.90
	Verrucomicrobiae					T6–T12	0.016	0.085	↓	–5.64	2.10
		Verrucomicrobiales				T0–T2	0.014	0.129	↑	5.03	1.70
		Verrucomicrobiales				T0–T3	0.021	0.135	↑	4.65	1.70
		Verrucomicrobiales				T0–T6	0.004	0.056	↑	6.30	1.90
		Verrucomicrobiales				T6–T12	0.016	0.108	↓	–5.63	2.11
			Akkermansiaceae			T0–T2	0.014	0.136	↑	5.02	1.69
			Akkermansiaceae			T0–T3	0.020	0.157	↑	4.66	1.69
			Akkermansiaceae			T0–T6	0.004	0.081	↑	6.32	1.90
			Akkermansiaceae			T6–T12	0.009	0.171	↓	–5.65	2.10
				Akkermansia		T0–T2	0.016	0.203	↑	4.72	1.63
				Akkermansia		T0–T3	0.024	0.204	↑	4.34	1.63
				Akkermansia		T0–T6	0.003	0.165	↑	5.91	1.81
				Akkermansia		T6–T12	0.018	0.247	↓	–5.25	2.01
Firmicutes	Clostridia	Clostridia_UCG.014				T0–T3	0.013	0.129	↑	1.87	0.62
Firmicutes	Clostridia	Clostridia_UCG.014				T0–T6	0.020	0.122	↑	3.46	1.36
Firmicutes	Clostridia	Clostridia_UCG.014				T6–T12	0.032	0.148	↓	–3.50	1.50
Firmicutes	Clostridia	Clostridia_UCG.014	Clostridia_UCG.014			T0–T3	0.012	0.136	↑	1.89	0.62
Firmicutes	Clostridia	Clostridia_UCG.014	Clostridia_UCG.014			T0–T6	0.020	0.173	↑	3.49	1.36
Firmicutes	Clostridia	Clostridia_UCG.014	Clostridia_UCG.014			T6–T12	0.030	0.193	↓	–3.54	1.51
Firmicutes	Clostridia	Clostridia_UCG.014	Clostridia_UCG.014	Clostridia_UCG.014		T0–T3	0.024	0.204	↑	1.70	0.64
Firmicutes	Clostridia	Clostridia_UCG.014	Clostridia_UCG.014	Clostridia_UCG.014	Genus_Clostridia_UCG.014	T0–T3	0.009	0.113	↑	2.21	0.68
Firmicutes	Clostridia	Clostridia_UCG.014	Clostridia_UCG.014	Clostridia_UCG.014	Genus_Clostridia_UCG.014	T0-T6	0.011	0.135	↑	3.58	1.25
Firmicutes	Clostridia	Clostridia_UCG.014	Clostridia_UCG.014	Clostridia_UCG.014	Genus_Clostridia_UCG.014	T6–T12	0.023	0.224	↓	–3.45	1.39
Firmicutes	Clostridia	Oscillospirales	Oscillospiraceae	UCG.005		T6–T12	0.009	0.171	↓	–2.80	0.96
Firmicutes	Clostridia	Oscillospirales	Oscillospiraceae	UCG.005	Genus_UCG.005	T0–T3	0.016	0.157	↑	2.54	0.87

Firmicutes	Clostridia	Oscillospirales	Oscillospiraceae	UCG.005	Genus_UCG.005	T0–T6	0.005	0.115	↑	2.66	0.84
Firmicutes	Clostridia	Oscillospirales	Oscillospiraceae	UCG.005	Genus_UCG.005	T6–T12	0.006	0.115	↓	−2.91	0.94
Firmicutes	Clostridia	Peptococcales				T0–T2	0.026	0.143	↑	2.24	0.85
Firmicutes	Clostridia	Peptococcales				T0–T6	0.069	0.207	↑	1.98	1.02
Firmicutes	Clostridia	Peptococcales				T6–T12	0.028	0.143	↓	−2.73	1.13
Firmicutes	Clostridia	Peptococcales	Peptococcaceae			T0–T2	0.025	0.175	↑	2.24	0.85
Firmicutes	Clostridia	Peptococcales	Peptococcaceae			T6–T12	0.026	0.184	↓	−2.76	1.13
Firmicutes	Clostridia	Peptococcales	Peptococcaceae	Family_Peptococcaceae		T0–T2	0.033	0.204	↑	2.06	0.83
Firmicutes	Clostridia	Peptococcales	Peptococcaceae		Family_Peptococcaceae	T0–T2	0.011	0.123	↑	2.73	0.88
Firmicutes	Clostridia	Peptococcales	Peptococcaceae		Family_Peptococcaceae	T0–T3	0.021	0.198	↑	2.41	0.88
Firmicutes	Clostridia	Peptococcales	Peptococcaceae		Family_Peptococcaceae	T0–T6	0.028	0.241	↑	2.10	0.87

b) Significant Increase Over Time in Several Microbial Taxa from Baseline up to T6 and stable relative abundance until T12

Phylum	Class	Order	Family	Genus	Species	Ref. Group	*p*	*q*	↓/↑	Coeff.	Std.Err.
Firmicutes	Clostridia	Christensenellales				T0–T2	0.010	0.129	↑	4.00	1.25
Firmicutes	Clostridia	Christensenellales				T0–T3	0.008	0.129	↑	4.14	1.25
Firmicutes	Clostridia	Christensenellales				T0–T6	0.005	0.056	↑	4.11	1.27
Firmicutes	Clostridia	Christensenellales	Christensenellaceae			T0–T2	0.010	0.131	↑	3.99	1.25
Firmicutes	Clostridia	Christensenellales	Christensenellaceae			T0–T3	0.008	0.131	↑	4.16	1.25
Firmicutes	Clostridia	Christensenellales	Christensenellaceae			T0–T6	0.004	0.081	↑	4.13	1.27
Firmicutes	Clostridia	Christensenellales	Christensenellaceae	Christensenellaceae_R.7_group		T0–T2	0.014	0.203	↑	2.16	0.72
Firmicutes	Clostridia	Christensenellales	Christensenellaceae	Christensenellaceae_R.7_group		T0–T3	0.011	0.203	↑	2.24	0.72
Firmicutes	Clostridia	Christensenellales	Christensenellaceae	Christensenellaceae_R.7_group		T0–T6	0.008	0.165	↑	2.12	0.71
Firmicutes	Clostridia	Christensenellales	Christensenellaceae	Christensenellaceae_R.7_group	Genus_Christensenellaceae_R.7_group	T0–T2	0.005	0.110	↑	2.57	0.72
Firmicutes	Clostridia	Christensenellales	Christensenellaceae	Christensenellaceae_R.7_group	Genus_Christensenellaceae_R.7_group	T0–T3	0.004	0.110	↑	2.70	0.72
Firmicutes	Clostridia	Christensenellales	Christensenellaceae	Christensenellaceae_R.7_group	Genus_Christensenellaceae_R.7_group	T0–T6	0.002	0.115	↑	2.58	0.71
Firmicutes	Clostridia	Oscillospirales	UCG.010			T0–T3	0.009	0.131	↑	1.26	0.39
Firmicutes	Clostridia	Oscillospirales	UCG.010			T0–T6	0.027	0.184	↑	1.69	0.70
Firmicutes	Clostridia	Oscillospirales	UCG.010			T2–T3	0.002	0.131	↑	1.60	0.39
Firmicutes	Clostridia	Oscillospirales	UCG.010	UCG.010		T0–T3	0.048	0.226	↑	1.05	0.46
Firmicutes	Clostridia	Oscillospirales	UCG.010	UCG.010		T2–T3	0.007	0.203	↑	1.57	0.46
Firmicutes	Clostridia	Oscillospirales	UCG.010	UCG.010	Genus_UCG.010	T0–T3	0.002	0.110	↑	1.62	0.39
Firmicutes	Clostridia	Oscillospirales	UCG.010	UCG.010	Genus_UCG.010	T2–T3	0.003	0.110	↑	1.55	0.39
Firmicutes	Clostridia	Oscillospirales	UCG.010	UCG.010	Genus_UCG.010	T0–T6	0.005	0.115	↑	1.98	0.62

c) Significant Alterations in the Microbial Taxa Abundance in the Short-Term Follow-up

Phylum	Class	Order	Family	Genus	Species	Ref. Group	*p*	*q*	↓/↑	Coeff.	Std.Err.
Firmicutes	Clostridia	Lachnospirales	Lachnospiraceae	X.Eubacterium._xylanophilum_group		T0–T2	0.046	0.226	↑	1.49	0.65
Firmicutes	Clostridia	Lachnospirales	Lachnospiraceae	X.Eubacterium._xylanophilum_group		T0–T3	0.002	0.203	↑	2.69	0.65
Firmicutes	Clostridia	Lachnospirales	Lachnospiraceae	X.Eubacterium._xylanophilum_group		T3–T6	0.016	0.235	↓	−3.05	1.16
Firmicutes	Clostridia	Lachnospirales	Lachnospiraceae	X.Eubacterium._xylanophilum_group	Genus_.Eubacterium._xylanophilum_group	T0–T2	0.009	0.113	↑	1.81	0.56
Firmicutes	Clostridia	Lachnospirales	Lachnospiraceae	X.Eubacterium._xylanophilum_group	Genus_.Eubacterium._xylanophilum_group	T0–T3	0.000	0.039	↑	3.06	0.56
Firmicutes	Clostridia	Lachnospirales	Lachnospiraceae	X.Eubacterium._xylanophilum_group	Genus_.Eubacterium._xylanophilum_group	T3–T6	0.022	0.224	↓	−2.89	1.16
Firmicutes	Clostridia	Lachnospirales	Lachnospiraceae	X.Eubacterium._eligens_group		T0–T2	0.018	0.203	↑	2.78	1.05
Firmicutes	Clostridia	Lachnospirales	Lachnospiraceae	X.Eubacterium._eligens_group		T0–T3	0.032	0.204	↑	2.49	1.05
Firmicutes	Clostridia	Lachnospirales	Lachnospiraceae	X.Eubacterium._eligens_group	Genus_.Eubacterium._eligens_group	T0–T2	0.005	0.110	↑	3.28	0.99
Firmicutes	Clostridia	Lachnospirales	Lachnospiraceae	X.Eubacterium._eligens_group	Genus_.Eubacterium._eligens_group	T0–T3	0.008	0.113	↑	3.03	0.99
Firmicutes	Clostridia	Oscillospirales	Oscillospiraceae	Intestinimonas		T0–T2	0.025	0.204	↑	2.69	1.02
Firmicutes	Clostridia	Lachnospirales	Lachnospiraceae	Lachnoclostridium		T0–T3	0.042	0.220	↓	−1.74	0.75
Firmicutes	Clostridia	Lachnospirales	Lachnospiraceae	Lachnoclostridium		T2–T3	0.006	0.203	↓	−2.57	0.75
Firmicutes	Clostridia	Lachnospirales	Lachnospiraceae	Lachnoclostridium	Genus_Lachnoclostridium	T2–T3	0.009	0.113	↓	−2.48	0.77
Firmicutes	Clostridia	Lachnospirales	Lachnospiraceae	Fusicatenibacter		T0–T2	0.016	0.203	↓	−2.12	0.73
Firmicutes	Clostridia	Lachnospirales	Lachnospiraceae	X.Ruminococcus._torques_group		T0–T3	0.041	0.220	↓	−1.40	0.60
Firmicutes	Clostridia	Lachnospirales	Lachnospiraceae	Dorea		T0–T2	0.032	0.204	↓	−1.85	0.74

d) Significantly Fluctuating Increase in Microbial Taxa in the Short-Term and Mid-Term Follow-ups

Phylum	Class	Order	Family	Genus	Species	Ref. Group	*p*	*q*	↓/↑	Coeff.	Std.Err.
Bacteroidota	Bacteroidia	Bacteroidales	Tannerellaceae			T0–T2	0.016	0.136	↑	1.71	0.59
Bacteroidota	Bacteroidia	Bacteroidales	Tannerellaceae			T2–T3	0.029	0.179	↓	−1.50	0.59
Bacteroidota	Bacteroidia	Bacteroidales	Tannerellaceae			T6–T12	0.002	0.080	↑	2.65	0.77
Bacteroidota	Bacteroidia	Bacteroidales	Tannerellaceae	Parabacteroides		T0–T2	0.043	0.220	↑	1.40	0.60
Bacteroidota	Bacteroidia	Bacteroidales	Tannerellaceae	Parabacteroides		T2–T3	0.027	0.204	↓	−1.57	0.60
Bacteroidota	Bacteroidia	Bacteroidales	Tannerellaceae	Parabacteroides		T6–T12	0.002	0.165	↑	2.82	0.88
Bacteroidota	Bacteroidia	Bacteroidales	Tannerellaceae	Parabacteroides	Parabacteroides_distasonis	T0–T2	0.007	0.113	↑	2.36	0.69
Bacteroidota	Bacteroidia	Bacteroidales	Tannerellaceae	Parabacteroides	Parabacteroides_distasonis	T2–T3	0.027	0.226	↓	−1.79	0.69
Bacteroidota	Bacteroidia	Bacteroidales	Tannerellaceae	Parabacteroides	Parabacteroides_distasonis	T6–T12	0.000	0.088	↑	3.72	0.88
Bacteroidota	Bacteroidia	Bacteroidales	Tannerellaceae	Parabacteroides	Parabacteroides_distasonis	T0–T12	0.008	0.115	↑	2.55	0.86
Proteobacteria	Gammaproteobacteria	Burkholderiales				T0–T12	0.046	0.173	↑	2.26	1.05
Proteobacteria	Gammaproteobacteria	Burkholderiales				T6–T12	0.039	0.159	↑	2.41	1.08

e) Significant Decrease in Some Microbial Taxa in the Mid-Term (at T6) Compared with Baseline											
**Phylum**	**Class**	**Order**	**Family**	**Genus**	**Species**	**Ref. Group**	***p***	***q***	**↓/↑**	**Coeff.**	**Std.Err.**
Desulfobacterota						T3–T6	0.012	0.074	↓	−2.72	0.98
Desulfobacterota						T6–T12	0.071	0.181	↑	2.08	1.08
Desulfobacterota	Desulfovibrionia					T3–T6	0.012	0.085	↓	−2.72	0.97
Desulfobacterota	Desulfovibrionia					T6–T12	0.070	0.188	↑	2.08	1.08
Desulfobacterota	Desulfovibrionia	Desulfovibrionales				T3–T6	0.012	0.101	↓	−2.72	0.97
Desulfobacterota	Desulfovibrionia	Desulfovibrionales				T6–T12	0.069	0.207	↑	2.08	1.07
Desulfobacterota	Desulfovibrionia	Desulfovibrionales	Desulfovibrionaceae			T3–T6	0.012	0.156	↓	−2.70	0.97
Desulfobacterota	Desulfovibrionia	Desulfovibrionales	Desulfovibrionaceae	Bilophila		T3–T6	0.006	0.165	↓	−2.75	0.88
Desulfobacterota	Desulfovibrionia	Desulfovibrionales	Desulfovibrionaceae	Bilophila		T0–T6	0.003	0.165	↓	−3.00	0.877
Desulfobacterota	Desulfovibrionia	Desulfovibrionales	Desulfovibrionaceae	Bilophila		T6–T12	0.006	0.165	↑	3.00	0.97
Desulfobacterota	Desulfovibrionia	Desulfovibrionales	Desulfovibrionaceae	Bilophila	Genus_Bilophila	T0–T6	0.008	0.115	↓	−2.49	0.83
Desulfobacterota	Desulfovibrionia	Desulfovibrionales	Desulfovibrionaceae	Bilophila	Genus_Bilophila	T3–T6	0.004	0.115	↓	−2.76	0.83
Desulfobacterota	Desulfovibrionia	Desulfovibrionales	Desulfovibrionaceae	Bilophila	Genus_Bilophila	T6–T12	0.003	0.115	↑	3.13	0.92
Firmicutes	Negativicutes					T0–T6	0.021	0.095	↓	−1.97	0.78
Firmicutes	Negativicutes					T3–T6	0.060	0.175	↓	−1.56	0.78
Firmicutes	Negativicutes					T6–T12	0.043	0.138	↑	1.88	0.86

f) Significant Decrease Over Time in Several Microbial Taxa up to T12											
**Phylum**	**Class**	**Order**	**Family**	**Genus**	**Species**	**Ref. Group**	***p***	***q***	**↓/↑**	**Coeff.**	**Std.Err.**
Firmicutes						T0–T2	0.009	0.120	↓	−0.74	0.23
Firmicutes						T0–T3	0.050	0.218	↓	−0.51	0.23
Firmicutes						T0–T6	0.001	0.019	↓	−0.97	0.24
Firmicutes						T0–T12	0.006	0.051	↓	−0.79	0.25
Firmicutes						T3–T6	0.072	0.181	↓	−0.46	0.24
Firmicutes	Clostridia					T0–T2	0.015	0.162	↓	−0.55	0.19
Firmicutes	Clostridia					T0–T6	0.043	0.138	↓	−0.68	0.31
Firmicutes	Clostridia					T0–T12	0.014	0.085	↓	−0.91	0.33
Firmicutes	Clostridia	Peptostreptococcales. Tissierellales				T0–T2	0.013	0.129	↓	−1.42	0.47
Firmicutes	Clostridia	Peptostreptococcales. Tissierellales				T0–T12	0.006	0.060	↓	−1.83	0.59
Firmicutes	Clostridia	Peptostreptococcales. Tissierellales				T6–T12	0.028	0.143	↓	−1.45	0.61
Firmicutes	Clostridia	Oscillospirales				T0–T2	0.019	0.135	↓	−0.85	0.30
Firmicutes	Clostridia	Oscillospirales				T0–T6	0.013	0.101	↓	−1.05	0.38
Firmicutes	Clostridia	Oscillospirales				T0–T12	0.056	0.186	↓	−0.84	0.411

Firmicutes	Clostridia	Oscillospirales	Ruminococcaceae			T0–T2	0.008	0.131	↓	−1.30	0.39
Firmicutes	Clostridia	Oscillospirales	Ruminococcaceae			T0–T6	0.002	0.081	↓	−1.66	0.47
Firmicutes	Clostridia	Oscillospirales	Ruminococcaceae			T0–T12	0.023	0.184	↓	−1.24	0.50
Firmicutes	Clostridia	Oscillospirales	Ruminococcaceae	Family_Ruminococcaceae		T0–T12	0.002	0.165	↓	−3.23	0.929
Firmicutes	Clostridia	Oscillospirales	Ruminococcaceae	Family_Ruminococcaceae	Family_Ruminococcaceae	T0–T12	0.007	0.115	↓	−2.61	0.86
Firmicutes	Clostridia	Lachnospirales				T0–T6	0.055	0.187	↓	−0.79	0.38
Firmicutes	Clostridia	Lachnospirales				T0–T12	0.003	0.056	↓	−1.40	0.41
Firmicutes	Clostridia	Lachnospirales	Lachnospiraceae	Agathobacter		T0–T2	0.013	0.203	↓	−2.65	0.87
Firmicutes	Clostridia	Lachnospirales	Lachnospiraceae	Agathobacter		T0–T6	0.012	0.191	↓	−3.25	1.18
Firmicutes	Clostridia	Lachnospirales	Lachnospiraceae	Agathobacter		T0–T12	0.008	0.165	↓	−3.67	1.26
Actinobacteriota						T0–T2	0.073	0.218	↓	−1.85	0.92
Actinobacteriota						T0–T3	0.087	0.224	↓	−1.75	0.92
Actinobacteriota						T6–T12	0.075	0.180	↓	−2.44	1.29
Actinobacteriota						T0–T12	0.020	0.081	↓	−3.19	1.25

g) Significant Alteration of Several Microbial Taxa in the Long-Term Follow-up (After T6) Compared with Baseline

Phylum	Class	Order	Family	Genus	Species	Ref. Group	*p*	*q*	↓/↑	Coeff.	Std.Err.
Firmicutes						T0–T12	0.006	0.051	↓	−0.79	0.25
Firmicutes	Clostridia					T0–T12	0.014	0.085	↓	−0.91	0.33
Firmicutes	Clostridia	Peptostreptococcales. Tissierellales				T0–T12	0.006	0.060	↓	−1.83	0.59
Firmicutes	Clostridia	Oscillospirales				T0–T12	0.056	0.186	↓	−0.84	0.411
Firmicutes	Clostridia	Oscillospirales	Ruminococcaceae			T0–T12	0.023	0.184	↓	−1.24	0.50
Firmicutes	Clostridia	Oscillospirales	Ruminococcaceae	Family_Ruminococcaceae		T0–T12	0.002	0.165	↓	−3.23	0.929
Firmicutes	Clostridia	Lachnospirales				T0–T12	0.003	0.056	↓	−1.40	0.41
Firmicutes	Clostridia	Lachnospirales	Lachnospiraceae	Agathobacter		T0–T12	0.008	0.165	↓	−3.67	1.26
Firmicutes	Clostridia	Lachnospirales	Lachnospiraceae			T0–T12	0.004	0.081	↓	−1.41	0.42
Firmicutes	Clostridia	Lachnospirales	Lachnospiraceae	Genus_.Eubacterium._hallii_group	Genus_.Eubacterium._hallii_group	T6–T12	0.017	0.196	↓	−2.73	1.02
Firmicutes	Clostridia	Oscillospirales	[Eubacterium]_coprostanoligenes_group	[Eubacterium]_coprostanoligenes_group	[Eubacterium]_coprostanoligenes_group	T6–T12	0.021	0.224	↓	−2.43	0.95
Actinobacteriota						T0–T12	0.020	0.081	↓	−3.19	1.25
Actinobacteriota						T6–T12	0.075	0.180	↓	−2.44	1.29
Actinobacteriota	Coriobacteriia					T0-T12	0.000	0.007	↓	−5.23	1.21
Actinobacteriota	Coriobacteriia					T6–T12	0.000	0.007	↓	−5.41	1.24
Actinobacteriota	Coriobacteriia	Coriobacteriales				T0–T12	0.000	0.012	↓	−5.26	1.20
Actinobacteriota	Coriobacteriia	Coriobacteriales				T6-T12	0.000	0.012	↓	−5.40	1.24
Actinobacteriota	Coriobacteriia	Coriobacteriales	Eggerthellaceae			T0–T12	0.020	0.173	↓	−3.30	1.28

Actinobacteriota	Coriobacteriia	Coriobacteriales	Eggerthellaceae			T6–T12	0.008	0.125	↓	−3.97	1.33
Bacteroidota	Bacteroidia	Bacteroidales	Tannerellaceae	Parabacteroides	Parabacteroides_distasonis	T0–T12	0.008	0.115	↑	2.55	0.86
Proteobacteria	Gammaproteobacteria	Burkholderiales				T0–T12	0.046	0.173	↑	2.26	1.05
**h) Significant Alteration of Several Microbial Taxa at Specific Time Points**											
	**Class**	**Order**	**Family**	**Genus**	**Species**	**Ref. Group**	***p***	***q***	**↓/↑**	**Coeff.**	**Std.Err.**
Bacteroidota						T0–T3	0.068	0.218	↓	−0.64	0.32
Bacteroidota						T6–T12	0.041	0.140	↑	0.780	0.35
Bacteroidota	Bacteroidia					T6–T12	0.039	0.138	↑	0.78	0.35
Bacteroidota	Bacteroidia	Bacteroidales				T0–T3	0.048	0.242	↓	−0.70	0.31
Bacteroidota	Bacteroidia	Bacteroidales				T6–T12	0.039	0.159	↑	0.78	0.35
Bacteroidota	Bacteroidia	Bacteroidales	Rikenellaceae	Alistipes		T0–T2	0.011	0.203	↓	−1.31	0.42
Bacteroidota	Bacteroidia	Bacteroidales	Rikenellaceae	Alistipes		T0–T3	0.032	0.204	↓	−1.05	0.42
Bacteroidota	Bacteroidia	Bacteroidales	Rikenellaceae	Alistipes	Alistipes_shahii	T0–T2	0.023	0.199	↓	−1.66	0.62
Bacteroidota	Bacteroidia	Bacteroidales	Rikenellaceae	Alistipes	Genus_Alistipes	T0-T6	0.026	0.239	↓	−0.96	0.40
Bacteroidota	Bacteroidia	Bacteroidales	Rikenellaceae	Alistipes	Genus_Alistipes	T6–T12	0.008	0.115	↑	1.31	0.44
Bacteroidota	Bacteroidia	Bacteroidales	Bacteroidaceae	Bacteroides		T0–T3	0.018	0.203	↓	−1.01	0.36
Bacteroidota	Bacteroidia	Bacteroidales	Bacteroidaceae	Bacteroides	Genus_Bacteroides	T6–T12	0.006	0.115	↑	4.76	1.51
Bacteroidota	Bacteroidia	Bacteroidales	Barnesiellaceae	Barnesiella		T0–T3	0.034	0.204	↓	−2.44	1.00
Bacteroidota	Bacteroidia	Bacteroidales	Marinifilaceae	Butyricimonas		T0–T3	0.041	0.220	↓	−1.51	0.64
Firmicutes	Clostridia	Oscillospirales	Ruminococcaceae	Subdoligranulum		T0–T2	0.019	0.203	↓	−1.88	0.67
Firmicutes	Clostridia	Lachnospirales	Lachnospiraceae	Anaerostipes		T0–T2	0.033	0.204	↓	−2.37	0.96

Statistical significance was evaluated by running a Generalized Linear Mixed-effects Model with MaAsLin2. Effect size is represented by the MaAsLin2 model coefficients (Coeff.) and respective standard errors (Std. Err.) Only taxa abundance changes at $p \leq 0.05$ and $q \leq 0.25$ are considered statistically significant. q: p adjusted for Benjamini–Hochberg (BH) correction test with a cut-off at $q \leq 0.25$. Samples were analyzed at baseline (T0), after two (T2), after three (T3), after six (T6) and after twelve (T12) months of the nutritional intervention. KETO= patients who followed a very-low-calorie ketogenic diet (VLCKD); Ref. group= time points compared; ↓= significantly reduced in the second term of the pairwise group; ↑= significantly increased in the second term of the pairwise group.

Table S15. Spearman correlation analysis between GM alterations and clinical and nutritional variables in patients with diabesity at baseline

Phylum	Class	Order	Family	Genus	Species	Variable	R	*p*
p_Actinobacteriota						Full fat dairy	-0,724	0,015
p_Actinobacteriota	c_Actinobacteria					MDS	-0,739	0,012
p_Actinobacteriota	c_Actinobacteria					Full fat dairy	-0,644	0,037
p_Actinobacteriota	c_Actinobacteria					Alcohol	-0,718	0,018
p_Actinobacteriota	c_Actinobacteria	o_Actinobacteria				MDS	-0,739	0,012
p_Actinobacteriota	c_Actinobacteria	o_Actinobacteria				Full fat dairy	-0,644	0,037
p_Actinobacteriota	c_Actinobacteria	o_Actinobacteria				Alcohol	-0,718	0,018
p_Actinobacteriota	c__Coriobacteriia	o__Coriobacteriales	f_Coriobacteriales_Incertae_Sedis			FM (%)	0,632	0,042
p_Actinobacteriota	c__Coriobacteriia	o__Coriobacteriales	f_Coriobacteriales_Incertae_Sedis			DBP (mmHg)	0,647	0,036
p_Actinobacteriota	c__Coriobacteriia	o__Coriobacteriales	f_Coriobacteriales_Incertae_Sedis			Food intake Kcal/day	-0,727	0,015
p_Actinobacteriota	c__Coriobacteriia	o__Coriobacteriales	f_Coriobacteriales_Incertae_Sedis			Carbohydrates %	0,626	0,042
p_Actinobacteriota	c__Coriobacteriia	o__Coriobacteriales	f_Coriobacteriales_Incertae_Sedis			Lipids %	-0,705	0,019
p_Actinobacteriota	c__Coriobacteriia	o__Coriobacteriales	f_Coriobacteriales_Incertae_Sedis	g_Family_Coriobacteriales_Incertae_Sedis		FM (%)	0,632	0,042
p_Actinobacteriota	c__Coriobacteriia	o__Coriobacteriales	f_Coriobacteriales_Incertae_Sedis	g_Family_Coriobacteriales_Incertae_Sedis		DBP (mmHg)	0,647	0,036
p_Actinobacteriota	c__Coriobacteriia	o__Coriobacteriales	f_Coriobacteriales_Incertae_Sedis	g_Family_Coriobacteriales_Incertae_Sedis		Food intake Kcal/day	-0,727	0,015
p_Actinobacteriota	c__Coriobacteriia	o__Coriobacteriales	f_Coriobacteriales_Incertae_Sedis	g_Family_Coriobacteriales_Incertae_Sedis		Carbohydrates %	0,626	0,042
p_Actinobacteriota	c__Coriobacteriia	o__Coriobacteriales	f_Coriobacteriales_Incertae_Sedis	g_Family_Coriobacteriales_Incertae_Sedis		Lipids %	-0,705	0,019
p_Actinobacteriota	c__Coriobacteriia	o__Coriobacteriales	f_Coriobacteriales_Incertae_Sedis	g_Family_Coriobacteriales_Incertae_Sedis	s_Family_Coriobacteriales_Incertae_Sedis	FM (%)	0,632	0,042
p_Actinobacteriota	c__Coriobacteriia	o__Coriobacteriales	f_Coriobacteriales_Incertae_Sedis	g_Family_Coriobacteriales_Incertae_Sedis	s_Family_Coriobacteriales_Incertae_Sedis	DBP (mmHg)	0,647	0,036
p_Actinobacteriota	c__Coriobacteriia	o__Coriobacteriales	f_Coriobacteriales_Incertae_Sedis	g_Family_Coriobacteriales_Incertae_Sedis	s_Family_Coriobacteriales_Incertae_Sedis	Food intake Kcal/day	-0,727	0,015
p_Actinobacteriota	c__Coriobacteriia	o__Coriobacteriales	f_Coriobacteriales_Incertae_Sedis	g_Family_Coriobacteriales_Incertae_Sedis	s_Family_Coriobacteriales_Incertae_Sedis	Carbohydrates %	0,626	0,042
p_Actinobacteriota	c__Coriobacteriia	o__Coriobacteriales	f_Coriobacteriales_Incertae_Sedis	g_Family_Coriobacteriales_Incertae_Sedis	s_Family_Coriobacteriales_Incertae_Sedis	Lipids %	-0,705	0,019
p_Actinobacteriota	c__Coriobacteriia	o__Coriobacteriales	f_Eggerthellaceae			FPG (mg/dl)	-0,733	0,013
p_Actinobacteriota	c__Coriobacteriia	o__Coriobacteriales	f_Eggerthellaceae			Red Meat	0,755	0,010
p_Bacteroidota	c__Bacteroidia	o__Bacteroidales	f_Bacteroidaceae			SF-36 MCS	-0,656	0,032
p_Bacteroidota	c__Bacteroidia	o__Bacteroidales	f_Bacteroidaceae			Fruit	0,630	0,043
p_Bacteroidota	c__Bacteroidia	o__Bacteroidales	f_Bacteroidaceae	g_Bacteroides		SF-36 MCS	-0,656	0,032

p_Bacteroidota	c__Bacteroidia	o__Bacteroidales	f_Bacteroidaceae	g_Bacteroides		Fruit	0,630	0,043
p_Bacteroidota	c__Bacteroidia	o__Bacteroidales	f_Tannerellaceae			Body weight (kg)	-0,752	0,010
p_Bacteroidota	c__Bacteroidia	o__Bacteroidales	f_Tannerellaceae			Daily sitting time (h/day)	-0,651	0,034
p_Bacteroidota	c__Bacteroidia	o__Bacteroidales	f_Tannerellaceae	s_Genus_Parabacteroides		Total Cholesterol (mg/dl)	-0,731	0,014
p_Bacteroidota	c__Bacteroidia	o__Bacteroidales	f_Tannerellaceae	s_Genus_Parabacteroides		LDL Cholesterol (mg/dl)	-0,791	0,006
p_Bacteroidota	c__Bacteroidia	o__Bacteroidales	f_Tannerellaceae	s_Genus_Parabacteroides		Daily sitting time (h/day)	-0,669	0,029
p_Bacteroidota	c__Bacteroidia	o__Bacteroidales	f_Tannerellaceae	g__Parabacteroides	s_Parabacteroides_distasonis	Body weight (kg)	-0,743	0,012
p_Bacteroidota	c__Bacteroidia	o__Bacteroidales	f_Tannerellaceae	g__Parabacteroides	s_Parabacteroides_distasonis	FFM (kg)	-0,679	0,026
p_Bacteroidota	c__Bacteroidia	o__Bacteroidales	f_Tannerellaceae	g__Parabacteroides	s_Parabacteroides_distasonis	Triglycerides (mg/dl)	-0,673	0,028
p_Bacteroidota	c__Bacteroidia	o__Bacteroidales	f__Marinifilaceae	g_Butyricimonas		Daily sitting time (h/day)	-0,821	0,003
p_Bacteroidota	c__Bacteroidia	o__Bacteroidales	f__Marinifilaceae	g_Butyricimonas	s_Genus_Butyricimonas	Daily sitting time (h/day)	-0,821	0,003
p_Bacteroidota	c__Bacteroidia	o__Bacteroidales	f__Barnesiellaceae	g__Barnesiella	s_Genus_Barnesiella	Total Cholesterol (mg/dl)	-0,735	0,013
p_Bacteroidota	c__Bacteroidia	o__Bacteroidales	f__Rikenellaceae	g__Alistipes	s_Alistipes_shahii	Total Cholesterol (mg/dl)	-0,671	0,028
p_Bacteroidota	c__Bacteroidia	o__Bacteroidales	f__Rikenellaceae	g__Alistipes	s_Alistipes_shahii	MDS	-0,691	0,022
p_Bacteroidota	c__Bacteroidia	o__Bacteroidales	f__Rikenellaceae	g__Alistipes	s_Alistipes_shahii	Daily sitting time (h/day)	-0,774	0,007
p_Desulfobacterota						MDS	0,662	0,030
p_Desulfobacterota						Alcohol	0,656	0,034
p_Desulfobacterota	c__Desulfovibrionia	o__Desulfovibrionales	f__Desulfovibrionaceae	g_Family_Desulfovibrionaceae		Body weight (kg)	-0,748	0,011
p_Desulfobacterota	c__Desulfovibrionia	o__Desulfovibrionales	f__Desulfovibrionaceae	g_Family_Desulfovibrionaceae		WC (cm)	-0,680	0,024
p_Desulfobacterota	c__Desulfovibrionia	o__Desulfovibrionales	f__Desulfovibrionaceae	g_Family_Desulfovibrionaceae		Non refined cereals	0,624	0,042
p_Desulfobacterota	c__Desulfovibrionia	o__Desulfovibrionales	f__Desulfovibrionaceae	g_Family_Desulfovibrionaceae	s_Family_Desulfovibrionaceae	Body weight (kg)	-0,748	0,011
p_Desulfobacterota	c__Desulfovibrionia	o__Desulfovibrionales	f__Desulfovibrionaceae	g_Family_Desulfovibrionaceae	s_Family_Desulfovibrionaceae	WC (cm)	-0,680	0,024
p_Desulfobacterota	c__Desulfovibrionia	o__Desulfovibrionales	f__Desulfovibrionaceae	g_Family_Desulfovibrionaceae	s_Family_Desulfovibrionaceae	Non refined cereals	0,624	0,042
p_Desulfobacterota	c__Desulfovibrionia	o__Desulfovibrionales	f__Desulfovibrionaceae	g_Desulfovibrio		HbA1C (%)	0,705	0,020
p_Desulfobacterota	c__Desulfovibrionia	o__Desulfovibrionales	f__Desulfovibrionaceae	g_Desulfovibrio	s_Genus_Desulfovibrio	phA (°)	-0,650	0,034
p_Desulfobacterota	c__Desulfovibrionia	o__Desulfovibrionales	f__Desulfovibrionaceae	g_Desulfovibrio	s_Genus_Desulfovibrio	HbA1C (%)	0,671	0,028
p_Desulfobacterota	c__Desulfovibrionia	o__Desulfovibrionales	f__Desulfovibrionaceae	g_Desulfovibrio	s_Genus_Desulfovibrio	Proteins %	-0,680	0,022
p_Desulfobacterota	c__Desulfovibrionia	o__Desulfovibrionales	f__Desulfovibrionaceae	g_Desulfovibrio	s_Genus_Desulfovibrio	Fruit	-0,681	0,020
p_Firmicutes	c_Clostridia					phA (°)	0,616	0,047
p_Firmicutes	c_Clostridia					SBP (mmHg)	-0,712	0,017

p_Firmicutes	c_Clostridia	o_Clostridia				phA (°)	0,616	0,047
p_Firmicutes	c_Clostridia	o_Clostridia				SBP (mmHg)	-0,712	0,017
p_Firmicutes	c_Clostridia	o__Clostridia_UCG-014	f_Clostridia_UCG-014			SBP (mmHg)	-0,693	0,022
p_Firmicutes	c_Clostridia	o__Clostridia_UCG-014	f_Clostridia_UCG-014			Red Meat	-0,749	0,011
p_Firmicutes	c_Clostridia	o__Clostridia_UCG-014	f_Clostridia_UCG-014	g_Clostridia_UCG-014		SBP (mmHg)	-0,693	0,022
p_Firmicutes	c_Clostridia	o__Clostridia_UCG-014	f_Clostridia_UCG-014	g_Clostridia_UCG-014		Red Meat	-0,749	0,011
p_Firmicutes	c_Clostridia	o__Clostridia_UCG-014	f_Clostridia_UCG-014	g_Clostridia_UCG-014		Poultry	-0,189	0,572
p_Firmicutes	c_Clostridia	o__Clostridia_UCG-014	f_Clostridia_UCG-014	g_Clostridia_UCG-014	s_Genus_Clostridia_UCG-014	SBP (mmHg)	-0,693	0,022
p_Firmicutes	c_Clostridia	o__Clostridia_UCG-014	f_Clostridia_UCG-014	g_Clostridia_UCG-014	s_Genus_Clostridia_UCG-014	Red Meat	-0,749	0,011
p_Firmicutes	c_Clostridia	o__Lachnospirales	f_Lachnospiraceae			phA (°)	0,767	0,008
p_Firmicutes	c_Clostridia	o__Lachnospirales	f_Lachnospiraceae			Fruit	0,887	0,001
p_Firmicutes	c_Clostridia	o__Lachnospirales	f_Lachnospiraceae	g_Lachnospiraceae_UCG-010		Triglycerides (mg/dl)	0,703	0,020
p_Firmicutes	c_Clostridia	o__Lachnospirales	f_Lachnospiraceae	g_Lachnospiraceae_UCG-010		PAL (METs/week)	-0,877	0,001
p_Firmicutes	c_Clostridia	o__Lachnospirales	f_Lachnospiraceae	g_Lachnospiraceae_UCG-010	s_Genus_Lachnospiraceae_UCG-010	Triglycerides (mg/dl)	0,703	0,020
p_Firmicutes	c_Clostridia	o__Lachnospirales	f_Lachnospiraceae	g_Lachnospiraceae_UCG-010	s_Genus_Lachnospiraceae_UCG-010	PAL (METs/week)	-0,877	0,001
p_Firmicutes	c_Clostridia	o__Lachnospirales	f_Lachnospiraceae	g_Dorea		Alcohol	0,773	0,009
p_Firmicutes	c_Clostridia	o__Lachnospirales	f_Lachnospiraceae	g_Anaerostipes		MDS	-0,625	0,044
p_Firmicutes	c_Clostridia	o__Lachnospirales	f_Lachnospiraceae	g_Anaerostipes	s_Genus_Anaerostipes	MDS	-0,625	0,044
p_Firmicutes	c_Clostridia	o__Lachnospirales	f_Lachnospiraceae	g_[Eubacterium]_hallii_group		Total Cholesterol (mg/dl)	-0,713	0,017
p_Firmicutes	c_Clostridia	o__Lachnospirales	f_Lachnospiraceae	g_[Eubacterium]_hallii_group		MDS	-0,747	0,011
p_Firmicutes	c_Clostridia	o__Lachnospirales	f_Lachnospiraceae	g_[Eubacterium]_hallii_group		Daily sitting time (h/day)	-0,741	0,012
p_Firmicutes	c_Clostridia	o__Lachnospirales	f_Lachnospiraceae	g_[Eubacterium]_hallii_group		Alcohol	-0,693	0,024
p_Firmicutes	c_Clostridia	o__Lachnospirales	f_Lachnospiraceae	g_[Eubacterium]_hallii_group	s_Genus_[Eubacterium]_hallii_group	Total Cholesterol (mg/dl)	-0,713	0,017
p_Firmicutes	c_Clostridia	o__Lachnospirales	f_Lachnospiraceae	g_[Eubacterium]_hallii_group	s_Genus_[Eubacterium]_hallii_group	MDS	-0,747	0,011
p_Firmicutes	c_Clostridia	o__Lachnospirales	f_Lachnospiraceae	g_[Eubacterium]_hallii_group	s_Genus_[Eubacterium]_hallii_group	Daily sitting time (h/day)	-0,741	0,012
p_Firmicutes	c_Clostridia	o__Lachnospirales	f_Lachnospiraceae	g_[Eubacterium]_hallii_group	s_Genus_[Eubacterium]_hallii_group	Alcohol	-0,693	0,024
p_Firmicutes	c_Clostridia	o__Lachnospirales	f_Lachnospiraceae	g_[Eubacterium]_eligens_group		LDL Cholesterol (mg/dl)	-0,725	0,016
p_Firmicutes	c_Clostridia	o__Lachnospirales	f_Lachnospiraceae	g_[Eubacterium]_eligens_group	s_Genus_[Eubacterium]_eligens_group	LDL Cholesterol (mg/dl)	-0,725	0,016
p_Firmicutes	c_Clostridia	o__Oscillospirales	f_[Eubacterium]_coprostanoligenes_group	g__[Eubacterium]_coprostanoligenes_group	s_Genus_[Eubacterium]_coprostanoligenes_group	Non refined cereals	0,615	0,049
p_Firmicutes	c_Clostridia	o__Lachnospirales	f_Lachnospiraceae	g_[Eubacterium]_xylanophilum_group		Legumes	0,729	0,015

p_Firmicutes	c_Clostridia	o__Lachnospirales	f_Lachnospiraceae	g__[Eubacterium]_xylanophilum_group	s_Genus_[Eubacterium]_xylanophilum_group	Legumes	0,729	0,015
p_Firmicutes	c_Clostridia	o__Lachnospirales	f_Lachnospiraceae	g_Lachnoclostridium		SF-36 PCS	-0,662	0,030
p_Firmicutes	c_Clostridia	o__Lachnospirales	f_Lachnospiraceae	g_Lachnoclostridium		Fish	-0,751	0,010
p_Firmicutes	c_Clostridia	o__Lachnospirales	f_Lachnospiraceae	g_Lachnoclostridium	s_Genus_Lachnoclostridium	SF-36 PCS	-0,662	0,030
p_Firmicutes	c_Clostridia	o__Lachnospirales	f_Lachnospiraceae	g_Lachnoclostridium	s_Genus_Lachnoclostridium	Fish	-0,751	0,010
p_Firmicutes	c_Clostridia	o__Lachnospirales	f_Lachnospiraceae	g_Agathobacter		FPG (mg/dl)	-0,738	0,012
p_Firmicutes	c_Clostridia	o__Lachnospirales	f_Lachnospiraceae	g_Agathobacter		Fruit	0,664	0,030
p_Firmicutes	c_Clostridia	o__Lachnospirales	f_Lachnospiraceae	g_Fusicatenibacter		FPG (mg/dl)	-0,781	0,006
p_Firmicutes	c_Clostridia	o__Lachnospirales	f_Lachnospiraceae	g_Fusicatenibacter		SF-36 PCS	-0,789	0,005
p_Firmicutes	c_Clostridia	o__Lachnospirales	f_Lachnospiraceae	g_Fusicatenibacter		Fish	-0,651	0,034
p_Firmicutes	c_Clostridia	o__Lachnospirales	f_Lachnospiraceae	g_Fusicatenibacter		Red Meat	0,665	0,029
p_Firmicutes	c_Clostridia	o__Oscillospirales	f_Ruminococcaceae			SBP (mmHg)	-0,665	0,029
p_Firmicutes	c_Clostridia	o__Oscillospirales	f_Ruminococcaceae			Full fat dairy	-0,633	0,041
p_Firmicutes	c_Clostridia	o__Oscillospirales	f_Ruminococcaceae	g_Family_Ruminococcaceae		HDL Cholesterol (mg/dl)	0,697	0,021
p_Firmicutes	c_Clostridia	o__Oscillospirales	f_Ruminococcaceae	g_Family_Ruminococcaceae		Legumes	0,687	0,023
p_Firmicutes	c_Clostridia	o__Oscillospirales	f_Ruminococcaceae	g_Family_Ruminococcaceae	s_Family_Ruminococcaceae	HDL Cholesterol (mg/dl)	0,697	0,021
p_Firmicutes	c_Clostridia	o__Oscillospirales	f_Ruminococcaceae	g_Family_Ruminococcaceae	s_Family_Ruminococcaceae	Legumes	0,687	0,023
p_Firmicutes	c_Clostridia	o__Oscillospirales	f_Ruminococcaceae	g_[Ruminococcus]_torques_group		SBP (mmHg)	-0,632	0,041
p_Firmicutes	c_Clostridia	o__Oscillospirales	f_Ruminococcaceae	g_[Ruminococcus]_torques_group	s_Genus_[Ruminococcus]_torques_group	phA (°)	0,636	0,040
p_Firmicutes	c_Clostridia	o__Oscillospirales	f_Ruminococcaceae	g_[Ruminococcus]_torques_group	s_Genus_[Ruminococcus]_torques_group	SBP (mmHg)	-0,657	0,032
p_Firmicutes	c_Clostridia	o__Oscillospirales	f_Ruminococcaceae	g_Subdoligranulum		FPG (mg/dl)	0,620	0,046
p_Firmicutes	c_Clostridia	o__Oscillospirales	f_Ruminococcaceae	g_Subdoligranulum	s_Genus_Subdoligranulum	FPG (mg/dl)	0,620	0,046
p_Firmicutes	c_Clostridia	o__Oscillospirales	f_Oscillospiraceae			LDL Cholesterol (mg/dl)	-0,664	0,031
p_Firmicutes	c_Clostridia	o__Oscillospirales	f_Oscillospiraceae	g_Family_Oscillospiraceae		phA (°)	0,685	0,024
p_Firmicutes	c_Clostridia	o__Oscillospirales	f_Oscillospiraceae	g_Family_Oscillospiraceae		Fruit	0,887	0,001
p_Firmicutes	c_Clostridia	o__Oscillospirales	f_Oscillospiraceae	g_Family_Oscillospiraceae	s_Family_Oscillospiraceae	phA (°)	0,685	0,024
p_Firmicutes	c_Clostridia	o__Oscillospirales	f_Oscillospiraceae	g_Family_Oscillospiraceae	s_Family_Oscillospiraceae	Fruit	0,887	0,001
p_Firmicutes	c_Clostridia	o__Oscillospirales	f_Oscillospiraceae	g_Intestinimonas		HDL Cholesterol (mg/dl)	0,803	0,005
p_Firmicutes	c__Clostridia	o__Christensenellales	f_Christensenellaceae			PAL (METs/week)	0,667	0,029
p_Firmicutes	c__Clostridia	o__Christensenellales	f_Christensenellaceae			Daily sitting time (h/day)	-0,821	0,003

p_Firmicutes	c__Clostridia	o__Christensenellales	f_Christensenellaceae			SF-36 MCS	-0,644	0,037
p_Firmicutes	c__Clostridia	o__Christensenellales	f_Christensenellaceae			Poultry	-0,648	0,036
p_Firmicutes	c__Clostridia	o__Christensenellales	f_Christensenellaceae	g_Family_Christensenellaceae		FPG (mg/dl)	-0,718	0,017
p_Firmicutes	c__Clostridia	o__Christensenellales	f_Christensenellaceae	g_Family_Christensenellaceae		SF-36 PCS	-0,635	0,041
p_Firmicutes	c__Clostridia	o__Christensenellales	f_Christensenellaceae	g_Family_Christensenellaceae		Red Meat	0,842	0,001
p_Firmicutes	c__Clostridia	o__Christensenellales	f_Christensenellaceae	g_Family_Christensenellaceae	s_Family_Christensenellaceae	FPG (mg/dl)	-0,718	0,017
p_Firmicutes	c__Clostridia	o__Christensenellales	f_Christensenellaceae	g_Family_Christensenellaceae	s_Family_Christensenellaceae	SF-36 PCS	-0,635	0,041
p_Firmicutes	c__Clostridia	o__Christensenellales	f_Christensenellaceae	g_Family_Christensenellaceae	s_Family_Christensenellaceae	Red Meat	0,842	0,001
p_Firmicutes	c__Clostridia	o__Christensenellales	f_Christensenellaceae	g_Christensenellaceae_R-7_group		Daily sitting time (h/day)	-0,630	0,042
p_Firmicutes	c__Clostridia	o__Christensenellales	f_Christensenellaceae	g_Christensenellaceae_R-7_group		Poultry	-0,725	0,015
p_Firmicutes	c__Clostridia	o__Christensenellales	f_Christensenellaceae	g_Christensenellaceae_R-7_group	s_Genus_Christensenellaceae_R-7_group	Daily sitting time (h/day)	-0,630	0,042
p_Firmicutes	c__Clostridia	o__Christensenellales	f_Christensenellaceae	g_Christensenellaceae_R-7_group	s_Genus_Christensenellaceae_R-7_group	Poultry	-0,725	0,015
p_Firmicutes	c__Clostridia	o__Peptococcales	f_Peptococcaceae			Fibers	0,641	0,038
p_Firmicutes	c__Clostridia	o__Peptococcales	f_Peptococcaceae			Full fat dairy	-0,635	0,040
p_Firmicutes	c__Clostridia	o__Peptococcales	f_Peptococcaceae	g_Family_Peptococcaceae		Fibers	0,641	0,038
p_Firmicutes	c__Clostridia	o__Peptococcales	f_Peptococcaceae	g_Family_Peptococcaceae		Full fat dairy	-0,635	0,040
p_Firmicutes	c__Clostridia	o__Peptococcales	f_Peptococcaceae	g_Family_Peptococcaceae	s_Family_Peptococcaceae	Fibers	0,641	0,038
p_Firmicutes	c__Clostridia	o__Peptococcales	f_Peptococcaceae	g_Family_Peptococcaceae	s_Family_Peptococcaceae	Full fat dairy	-0,635	0,040
p_Firmicutes	c_Negativicutes					Non refined cereals	-0,633	0,041
p_Firmicutes	c_Negativicutes					Fish	-0,664	0,030
p_Firmicutes	c_Negativicutes	o_Negativicutes				Non refined cereals	-0,633	0,041
p_Firmicutes	c_Negativicutes	o_Negativicutes				Fish	-0,664	0,030
p_Verrucomicrobiota						SF-36 PCS	0,677	0,026
p_Verrucomicrobiota						Carbohydrates %	-0,718	0,016
p_Verrucomicrobiota						Vegetables	0,680	0,027
p_Verrucomicrobiota						Red Meat	-0,706	0,019
p_Verrucomicrobiota	c_Verrucomicrobiae					Carbohydrates %	-0,677	0,026
p_Verrucomicrobiota	c_Verrucomicrobiae					Lipids %	0,638	0,039
p_Verrucomicrobiota	c_Verrucomicrobiae					Vegetables	0,816	0,005
p_Verrucomicrobiota	c_Verrucomicrobiae	o_Verrucomicrobiae				Carbohydrates %	-0,677	0,026

p_Verrucomicrobiota	c_Verrucomicrobiae	o_Verrucomicrobiae				Lipids %	0,638	0,039
p_Verrucomicrobiota	c_Verrucomicrobiae	o_Verrucomicrobiae				Vegetables	0,816	0,005
p_Verrucomicrobiota	c_Verrucomicrobiae	o__Verrucomicrobiales	f_Akkermansiaceae			Carbohydrates %	-0,677	0,026
p_Verrucomicrobiota	c_Verrucomicrobiae	o__Verrucomicrobiales	f_Akkermansiaceae			Lipids %	0,638	0,039
p_Verrucomicrobiota	c_Verrucomicrobiae	o__Verrucomicrobiales	f_Akkermansiaceae			Vegetables	0,816	0,005
p_Verrucomicrobiota	c_Verrucomicrobiae	o__Verrucomicrobiales	f_Akkermansiaceae	g_Akkermansia		Carbohydrates %	-0,677	0,026
p_Verrucomicrobiota	c_Verrucomicrobiae	o__Verrucomicrobiales	f_Akkermansiaceae	g_Akkermansia		Lipids %	0,638	0,039
p_Verrucomicrobiota	c_Verrucomicrobiae	o__Verrucomicrobiales	f_Akkermansiaceae	g_Akkermansia		Vegetables	0,816	0,005
p_Verrucomicrobiota	c_Verrucomicrobiae	o__Verrucomicrobiales	f_Akkermansiaceae	g_Akkermansia	s_Akkermansia_muciniphila	SF-36 PCS	0,657	0,033
p_Verrucomicrobiota	c_Verrucomicrobiae	o__Verrucomicrobiales	f_Akkermansiaceae	g_Akkermansia	s_Akkermansia_muciniphila	Carbohydrates %	-0,675	0,027
p_Verrucomicrobiota	c_Verrucomicrobiae	o__Verrucomicrobiales	f_Akkermansiaceae	g_Akkermansia	s_Akkermansia_muciniphila	Vegetables	0,666	0,031
p_Verrucomicrobiota	c_Verrucomicrobiae	o__Verrucomicrobiales	f_Akkermansiaceae	g_Akkermansia	s_Akkermansia_muciniphila	Red Meat	-0,670	0,029

Spearman’s correlations were calculated in GraphPad Prism software v.7.0d. Only significant bacterial taxa identified in the Generalized Linear Mixed-effects Model were correlated to clinical and nutritional parameters. R= r coefficient (Rho), Rho > 0.5= positive correlation, Rho < − 0.5= negative correlation, *p*-value equal to or less than 0.05 was considered statistically significant. * = $p \leq 0.05$; ** = $p \leq 0.01$; *** = $p \leq 0.001$. BMI = Body Mass Index, WC = Waist Circumference, FM (%)= Fat Mass expressed in percentage, FFM; Free Fat Mass expressed in kilograms, phA (°)= phase Angle, FPG = fasting plasma glucose, HbA1c = glycosylated hemoglobin, SBP = Systolic Blood Pressure, DBP = Diastolic Blood Pressure, MDS = Mediterranean Diet Score, PAL = Physical Activity Level, METs/week = Metabolic Equivalent of Task-minutes per week, SF-36 PCS = Physical Component Summary of SF-36, SF-36 MCS = Mental Component Summary of SF-36.

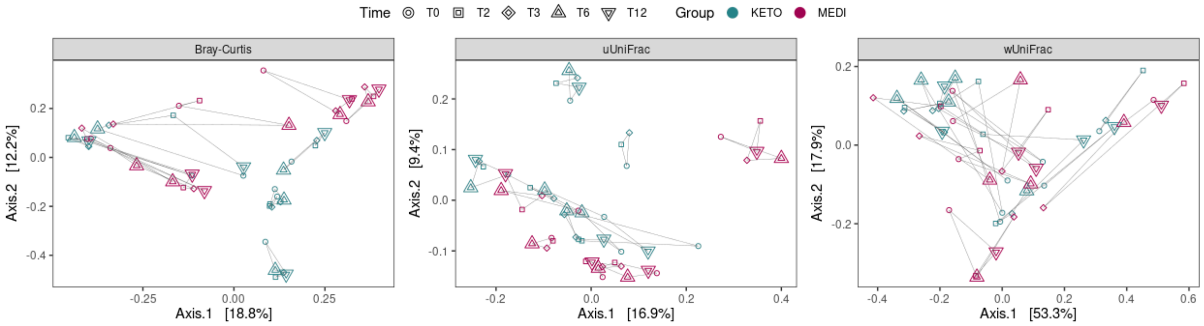

Figure S1. Principal coordinate analysis plots showing gut microbiota compositional changes along time points for each diet. Each subplot concerns a different beta diversity metric (Bray-Curtis, unweighted UniFrac, or weighted UniFrac). Samples from the same patient are linked by a gray line. KETO= patients who followed a very-low-calorie ketogenic diet (VLCKD), MEDI= patients who followed a low-calorie Mediterranean diet (MD). Samples were analyzed at baseline (T0), after two months (T2), after three months (T3), after six months (T6) and after twelve months (T12) of nutritional intervention.

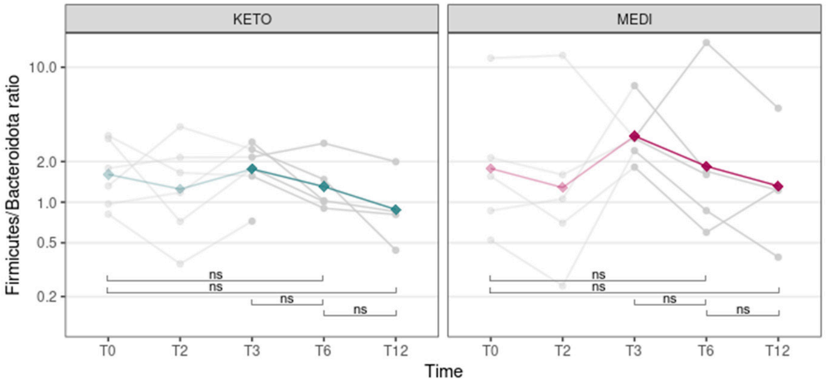

Figure S2. Gut microbiota Firmicutes/Bacteoroidota ratio comparison between time points for each diet. Each subplot concerns a different diet (KETO or MEDI). Samples from the same patient are linked by a gray line. The colored lines depict the mean values at each time point. pale colored plot= results up to T3 follow-up already published [1]; bright colored line= new results relating to analyzes extended up to T12. A $\log_{10}$ y-axis was used. Statistical significance was evaluated by the paired Wilcoxon signed-rank test and it was indicated as follows: ns, non-significant. p equal to or less than 0.05 was considered statistically significant. KETO = patients who followed a very-low-calorie ketogenic diet (VLCKD), MEDI = patients who followed a low-calorie Mediterranean diet (MD). Samples were analyzed at baseline (T0), after two months (T2), after three months (T3), after six months (T6) and after twelve months (T12) of nutritional intervention.

a) KETO

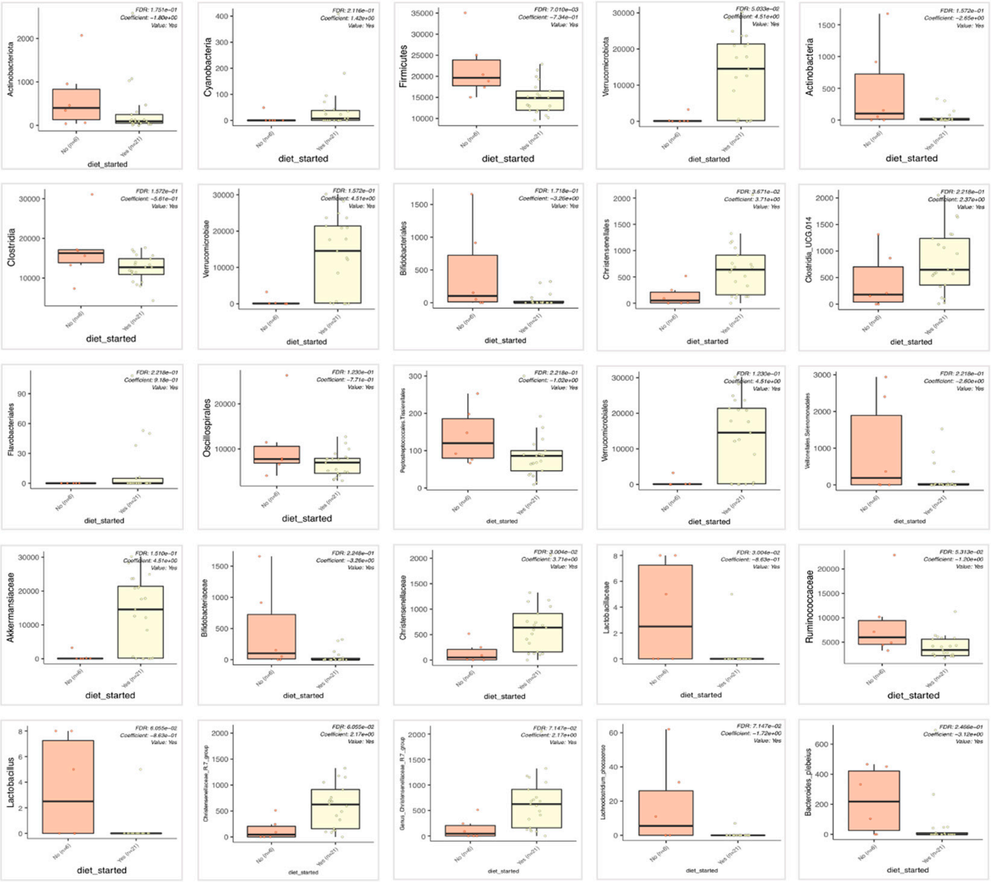
Actinobacteriota
Cyanobacteria
Firmicutes
Verrucomicrobiota
Actinobacteria
Clostridia
Verrucomicrobiae
Bifidobacteriales
Christensenellales
Clostridia_UCG.014
Flavobacteriales
Oscillospirales
Verrucomicrobiales
Akkermansiaceae
Bifidobacteriaceae
Christensenellaceae
Lactobacillaceae
Ruminococcaceae
Lactobacillus
Bacteroides_plebeius
diet_started

b) MEDI

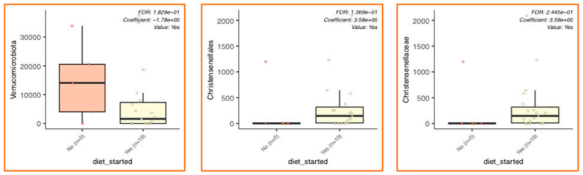
Verrucomicrobiota
Christensenellales
Christensenellaceae
diet_started

Figure S3. Significant changes in gut microbiota taxa abundance between time points in KETO and MEDI cohorts. Each subplot concerns a comparison between baseline (No diet started) and after the initiation of the diet (Yes diet started, with repeated sampling from the same individuals) in KETO (a) and MEDI (b) cohorts. Statistical significance was evaluated by running a Generalized Linear Mixed-effects Model with MaAsLin2 with individual as random effect. Effect size is presented in each subplot as the MaAsLin2 model coefficient and graphically through a boxplot of normalized counts. Only taxa abundance changes at $p \leq 0.05$ and q (FDR) ≤ 0.25 are considered statistically significant. q: p-valued adjusted with Benjamini–Hochberg (BH) correction. KETO = patients who followed a very-low-calorie ketogenic diet (VLCKD), MEDI = patients who followed a low-calorie Mediterranean diet (MD).

a)

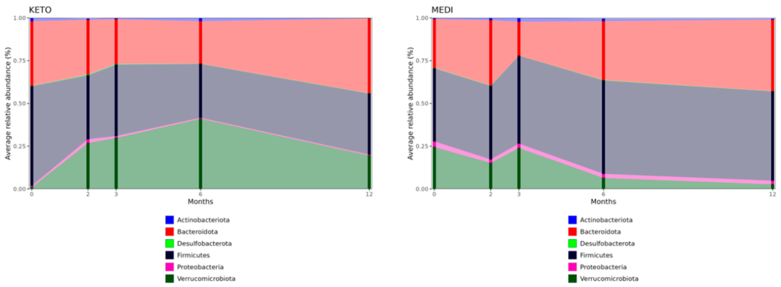

b)

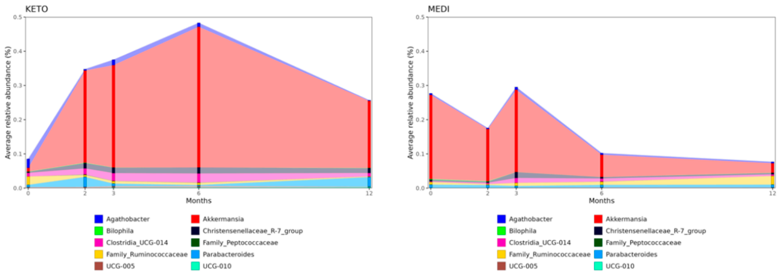

c)

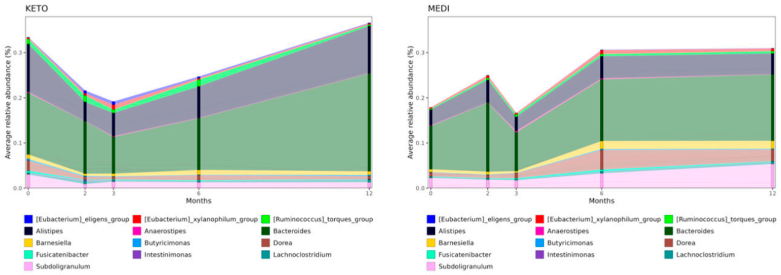

Figure S4. Graphical changes in gut microbiota taxa abundance over time in KETO and MEDI groups. Some of the significant data observed in KETO were graphically represented at phylum (a) and genus (b and c) levels for both KETO and MEDI groups. Samples were represented at baseline (T0) and after two (T2), three (T3), six (T6) and twelve (T12) months of NI. KETO= patients who followed a very-low-calorie ketogenic diet (VLCKD), MEDI= patients who followed a low-calorie Mediterranean diet (MD). No significant results in MEDI at mid-term and long-term follow-ups were observed; few significant results in MEDI at the short-term follow-up were observed (see previously published data [1]).

References

1. Deledda A., Palmas V., Heidrich V., Fosci M., Lombardo M., Cambarau G., Lai A., Melis M., Loi E., Loviselli A., Manzin A., Velluzzi F. Dynamics of Gut Microbiota and Clinical Variables after Ketogenic and Mediterranean Diets in Drug-Naïve Patients with Type 2 Diabetes Mellitus and Obesity. *Metabolites*. **2022** Nov 10;12(11):1092. doi: 10.3390/metabo12111092
